# Mutations in sigma 70 transcription factor improves expression of functional eukaryotic membrane proteins in *Escherichia coli*

**DOI:** 10.1038/s41598-019-39492-9

**Published:** 2019-02-21

**Authors:** Pablo Emiliano Tomatis, Marco Schütz, Elina Umudumov, Andreas Plückthun

**Affiliations:** 1 Department of Biochemistry, University of Zurich, Winterthurerstrasse 190, CH-8057 Zurich, Switzerland; 2 Present Address: Instituto de Biología Molecular y Celular de Rosario (IBR, CONICET-UNR), Ocampo y Esmeralda, 2000 Rosario, Argentina; 3 Present Address: Heptares Therapeutics Zürich AG, Schlieren, Grabenstrasse 11a, CH-8952 Schlieren, Switzerland; 4 Present Address: Janssen-Cilag AG, Hochstrasse 201, CH-8200 Schaffhausen, Switzerland

## Abstract

Eukaryotic integral membrane proteins (IMPs) are difficult to study due to low functional expression levels. To investigate factors for efficient biogenesis of eukaryotic IMPs in the prokaryotic model organism *Escherichia coli*, important, e.g., for isotope-labeling for NMR, we selected for *E. coli* cells expressing high levels of functional G protein-coupled receptors (GPCRs) by FACS. Utilizing an *E. coli* strain library with all non-essential genes systematically deleted, we unexpectedly discovered upon whole-genome sequencing that the improved phenotype was not conferred by the deleted genes but by various subtle alterations in the “housekeeping” sigma 70 factor (RpoD). When analyzing effects of the *rpoD* mutations at the transcriptome level we found that toxic effects incurred on wild-type *E. coli* during receptor expression were diminished by two independent and synergistic effects: a slower but longer-lasting GPCR biosynthesis and an optimized transcriptional pattern, augmenting growth and expression at low temperature, setting the basis for further bacterial strain engineering.

## Introduction

Integral membrane proteins (IMPs) have many vital biological functions, constituting approximately one third of all proteins in humans as well as being the targets of nearly 60% of all FDA-approved drugs^1,2^. Despite their importance, structural and functional information for IMPs is still limited. Up to now, fewer than 4% of the unique structures in the protein database (PDB) correspond to membrane proteins, and even fewer are of eukaryotic origin. For many of them, in particular for G protein-coupled receptors (GPCRs), there are no homologs from prokaryotes. GPCRs, the biggest IMP family, constitute almost 5% of the entire protein-coding human genome and are the most important class of drug targets.

Apart from a few exceptions, most IMPs have an extremely low natural abundance. Thus, they need to be overexpressed in heterologous hosts for detailed investigations^2–4^. Next to microbial hosts, such as *Escherichia coli*, *Lactococcus* or different yeast species (e.g., *Pichia pastoris* and *Saccharomyces cerevisiae*), researchers have been using mammalian cell cultures, insect cells, or cell-free systems for heterologous expression of IMPs, each with various degrees of success^4–6^. *E. coli* is a particularly attractive expression host because of its cost-effective cultivation and fast growth and especially its ability to produce isotope-labeled protein for NMR studies^7^ and rapid protein engineering approaches, by rational and combinatorial means.

Since many IMPs are very unstable when solubilized in detergents, different approaches based on either rational design^4,8^ or random mutagenesis and screening^9^ were used to obtain better expressing and more stable variants of IMPs. With the goal to improve heterologous expression of GPCRs and to create receptor variants with increased stability, our lab has developed several directed evolution strategies in *E. coli* and yeast^10–14^. In the present study, however, we focus on the bacterial host itself.

In most cases, eukaryotic hosts tolerate the heterologous overexpression of IMPs better than bacteria. While bacteria are able to produce some of their endogenous membrane proteins in high abundance, many IMPs, especially those of eukaryotic origin, are very toxic for the bacterial cell when overexpressed. Since polypeptide elongation is significantly slower in eukaryotes than in prokaryotes^4^, the overexpression of eukaryotic IMPs in bacteria may cause mistargeting and misfolding not only of the IMP itself, but also of other proteins, leading to high cellular stress. Furthermore, the titration of the Sec translocon, the limited availability of other endogenous factors assisting in the biogenesis of membrane proteins, or differences in membrane bilayer properties and membrane space can all affect insertion, folding and functioning of heterologous IMPs^15,16^.

In this study, we aimed to improve our understanding of heterologous expression of IMPs in *E. coli*, which would potentially allow the generation of bacterial expression strains that are more tolerant to the overexpression of eukaryotic IMPs. While for crystallography and electron microscopy, protein production can be carried out in insect cells or mammalian cells, for NMR, the facile preparation of isotope-labeled IMPs in *E. coli* would be a great advantage.

We planned to elucidate the bottlenecks of eukaryotic IMP biogenesis in bacteria with an unbiased approach, not limiting ourselves to a particular pathway, using pharmacologically relevant GPCRs as a model system. It was our aim to solve this problem not only practically, but also to make a contribution to elucidating the mechanism. Currently, the knowledge of the basic cellular processes that govern the biogenesis of heterologous IMPs in bacteria remains incomplete, and a systematic characterization of bacterial genes involved in this process as well as possible epistatic genetic interactions between them is still lacking. It seems intuitive that all steps during the biogenesis of a membrane protein need to be extremely well coordinated and balanced^17^. As a consequence, a rational approach to improve an IMP protein production system that finally would lead to higher levels of functionally expressed IMPs would be very difficult.

For this reason, we used a methodology previously developed in our laboratory that allows the detection of functional IMPs in *E. coli* at the single cell level by using fluorescent ligands and FACS, thus being able to select individual cells with improved functional expression level. As a model system, we used GPCRs to be expressed in *E. coli*. Our approach finally led to the discovery that a specifically mutated sigma factor has favorable effects at this process at several levels.

## Results

### Selection from the Keio collection for improved GPCR expression

#### Selection

In order to identify *E. coli* genes that could affect the heterologous production of GPCRs, we used a selection strategy based on fluorescence-activated cell sorting (FACS) developed in our laboratory that allows the isolation of cells showing increased expression levels of functional receptors that bind to a fluorescent ligand^10–12^. We screened a systematically constructed *E. coli* strain library consisting of clones, each with a precisely defined single-gene deletion in the genome, the so-called Keio collection^18^. We searched for *E. coli* strains showing increased functional expression of the rather unstable wild-type rat neurotensin 1 receptor (NTR1), which puts a significant stress on the host^10^.

The pooled Keio collection, consisting of 3985 different strains, was transformed with an expression vector denoted as pRG-NTR, encoding NTR1^10^. The transformation efficiency was 3 × 10^6^ cfu, well 100-fold above the library size to ensure exhaustive representation, giving rise to the *E. coli* strain library termed Keio-NTR1. After expression of NTR1 at 20 °C, permeabilization of the outer membrane and incubation of the cells with a fluorescently-labeled neurotensin ligand allowed it to bind to the functional receptor in the inner membrane of *E. coli* as previously described^10^. Subsequently, the most fluorescent cells, which consequently exhibit the highest expression levels of functional receptor, were isolated with FACS (Fig. S1).

After six iterative selection rounds, we could not detect an improvement of the expression level within the strain library expressing NTR1 (Fig. S2), as under these conditions of limited growth the fluorescence signal was similar between the pool of the selected *E. coli* deletion clones compared to the corresponding wild-type *E. coli* strain. However, we noticed variations in the fluorescence signal within experiments for some tested strains, so if the upshift of the fluorescence signal in the sorted clones is small and counterbalanced by strains in the pool with decreased receptor expression, a positive effect in the signal may go undetected. Therefore, we decided to investigate whether the selections may still have resulted in an enrichment of certain Keio clones over others, e.g., because the enriched clones may tolerate the expression of NTR1 better than the wild-type strain and may thus harbor favorable properties.

#### Identification and characterization of selected strains

The identification by inverse PCR of the selected *E. coli* Keio strains revealed that five specific clones from the Keio collection had indeed become enriched (Fig. S3). Intriguingly, there was no obvious direct relationship of the function of the genes that are deleted on these enriched clones with known pathways of membrane protein biosynthesis.

These five enriched strains from the selection were picked again from the original Keio collection, thus not harboring any plasmid, for further analysis. A characterization of the growth behavior in rich medium (2 × YT medium) in comparison to the control strain BW25113, from which the Keio library was derived, showed that the growth rates and the final cell density after 20 hours of cultivation at 37 °C were identical. On the other hand, the final cell density reached after 20 hours of cultivation at 20 °C was higher for 4 of 5 clones (Fig. S4), even though their growth rates at 20 °C were equivalent.

These Keio *E. coli* strains as well as the control strain BW25113 were then freshly transformed with the vector pRG-NTR. The presence of the corresponding gene deletions in the freshly transformed strains was verified by iPCR and sequencing.

**Figure 1:**
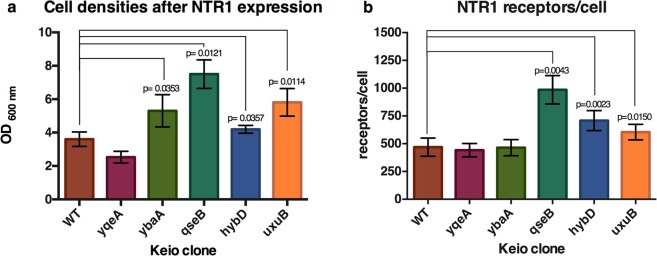
Expression of NTR1 receptor in the selected *E. coli* strains from the Keio collection. *E. coli* strain BW25113 (WT) and the most abundant clones of the selected Keio clones were transformed with the plasmid pRG-NTR. Expressions of NTR1 receptor are as described in Materials and Methods. The x-axis label indicates the gene that is deleted on the respective Keio clone. Means and Standard Deviations from three independent experiments are shown. (**a**) Growth was estimated with OD_600nm_ measurements after 20 hours of GPCR expression at 20 °C. Strains with statistically significant increases in growth versus wild-type *E. coli* BW25113 are calculated by two-tailed paired *t* test; *p* values are indicated. (**b**) Receptor expression levels were assessed by radioligand binding assays. The y-axis represents the number of functional receptors per cell, averaged across an entire population of cells. Statistical *p* values, as calculated by two-tailed paired *t* test, are indicated for strains with statistically significant increases in receptors per cell versus wild-type *E. coli* BW25113.

Following identical culture conditions and protocol for receptor expression as in the selection procedure, we found that the same selected Keio strains (*ΔybaA, ΔqseB, ΔhybD and ΔuxuB*) expressing NTR1 can also reach a higher final cell density after 20 hours at 20 °C, in comparison to the wt *E. coli* strain BW25113 (Fig. 1), all having the same growth rates. At 20 °C, the optimal temperature for functional receptor expression and selection, the overexpression of GPCRs is not quite as stressful for the cells as at 37 °C^10^, and apparently these Keio strains can grow for a more extended duration than the strain BW25113.

As expected from previous experiments, expression of the GPCRs is clearly toxic for the host, as the untransformed strains (without NTR1 expressed) reach higher cell densities than with NTR1 (Figs 1 and S4).

We then investigated whether the production of functional receptors was affected in addition to the improved growth behavior discovered. Indeed, although GPCR expression measurements are usually noisy, a statistically significant increase in functional receptors/cell was found for NTR1 measured by radioligand binding assays (RLBA) in the selected Keio clones *ΔqseB, ΔhybD and ΔuxuB* (Fig. 1).

### No genotype - phenotype link with Keio deletions

In order to check our findings, we re-constructed by recombineering^19^ the equivalent single-gene deletion clones from the Keio collection in the same genetic background of the control strain BW25113.

Unexpectedly, the growth and receptor levels in these newly made deletion strains were as in the control strain. Thus, there seems to be no direct correlation between the deleted genes and the observed phenotype of improved GPCR expression.

While the enrichment of the original Keio clones (*ΔhybD*, *ΔqseB*, *ΔybaA* and *ΔuxuB*) was reproducible, as were their phenotypes regarding resistance to GPCR expression stress, it appeared that these properties were not related to the presumed genotypes denoted in the Keio collection, but still somehow linked to the particular strains.

### Identification of causative mutation by whole genome sequencing

To identify the genetic modification that causes the difference in the observed phenotype, the DNA sequence of the whole genome of the most promising selected Keio clone (*ΔqseB*) and the wild-type strain BW25113 were determined. The obtained genome sequence of BW25113 is in agreement with a sequence deposited afterwards (GenBank accession number CP009273)^20^.

**Figure 2:**
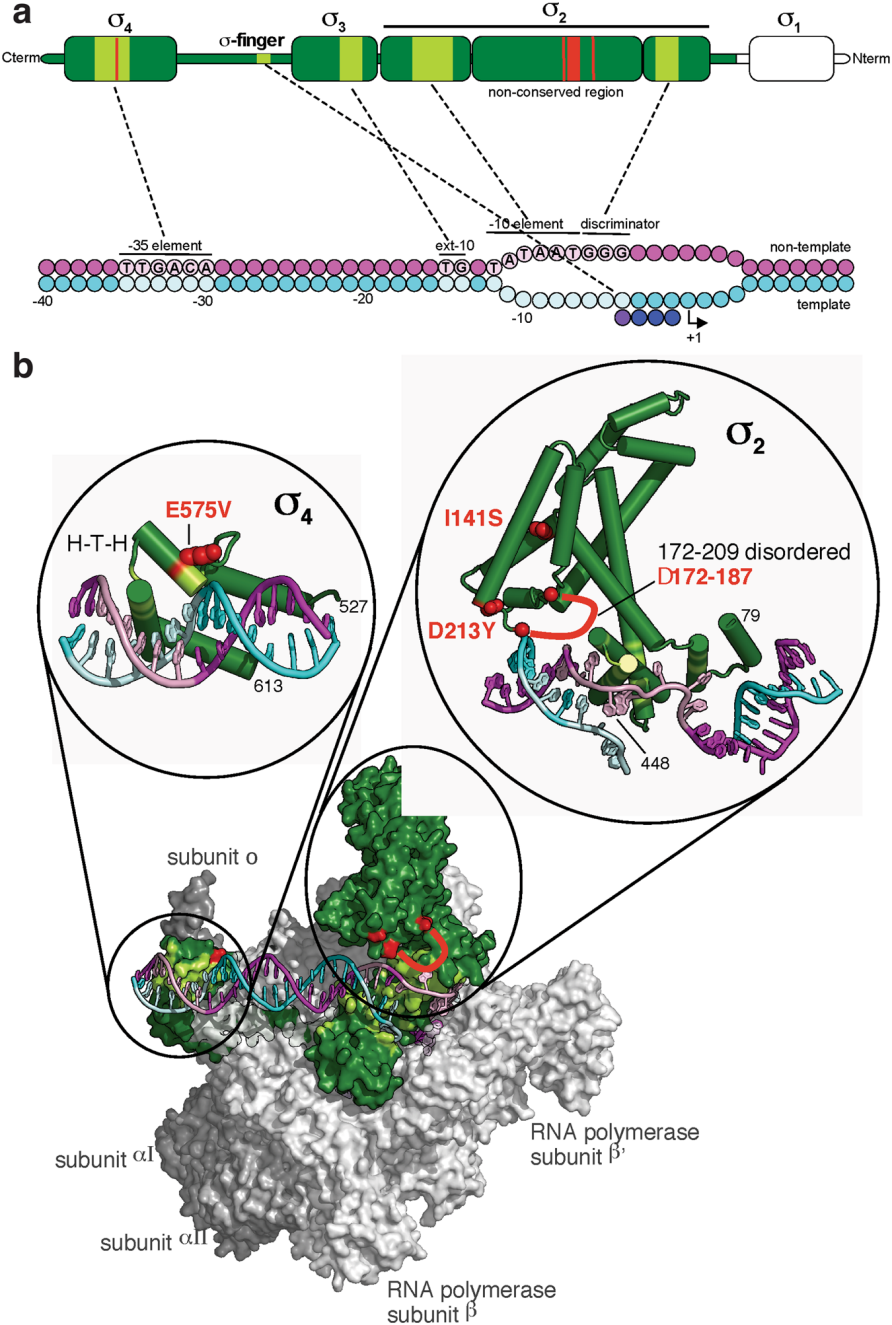
Structural interactions of sigma 70 factor in the RNA polymerase transcription initiation complex and location of selected mutations. (**a**) Domain organization, conserved regions and promoter recognition of the sigma 70 factor (green). Interactions between sigma 70 and DNA are depicted with dashed lines. The non-template DNA strand is colored magenta and the template strand cyan, with key consensus promoter elements contacted by sigma 70 (light green) indicated in pink with letters. Transcription initiates at +1. Mutations found in the *rpoD* gene in this work are highlighted in red. Adapted from^65^. (**b**) Overall structure and organization of *E. coli* sigma 70 in the RNA polymerase transcription initiation complex based on PDB ID: 4YLN^39^. Sigma 70 (surface representation) and promoter DNA (cartoon) are colored as above. Inset zooms show the HTH domain of sigma 70 where the mutation E575V is located, and interaction of mutations in sigma 70 domain 2 with DNA. Adapted from^65^.

In the *ΔqseB* Keio clone, a non-silent single nucleotide substitution in the *rpoD* gene was identified and confirmed by Sanger sequencing. This essential gene encodes the sigma 70 subunit of the RNA polymerase, the primary sigma factor during exponential growth, which targets RNA polymerase to the majority of promoters operative under normal growth conditions^21^. The nucleotide change in the *rpoD* gene results in a valine instead of glutamate in amino acid position 575 (domain 4.2) of the sigma 70 transcription factor (Fig. 2). This position plays an important role in the interaction between RNA polymerase and promoters^22^ (Fig. 2).

### Recreating new *E. coli* strains with and without the *rpoD* mutation

To confirm the correlation of this mutation with the observed phenotype we wanted to create a new *E. coli* strain carrying only the mutation *rpoD*-E575V. For this purpose, a site-directed genome mutagenesis approach based on recombineering was developed (detailed in Supplementary Information: Supplementary Methods). Briefly, we created two DNA fragments comprising the kanamycin resistance cassette targeted to the *mug* gene, downstream of *rpoD* (encoding the non-essential mismatch-specific uracil DNA-glycosylase) and the last 350 bp of the *rpoD* gene with the E575V mutation or, alternatively, with the wild-type sequence (Fig. S5). Using recombineering^19^ we thus generated two new *E. coli* strains: BW25113 *rpoD-*E575V *Δmug::Km* (abbreviated rpoD* from now on) and the control strain BW25113 wt *rpoD(E575) Δmug::Km*, which is *rpoD wt* (Fig. S5). These two newly constructed strains thus differ only by the E575V mutation in *rpoD*.

**Figure 3:**
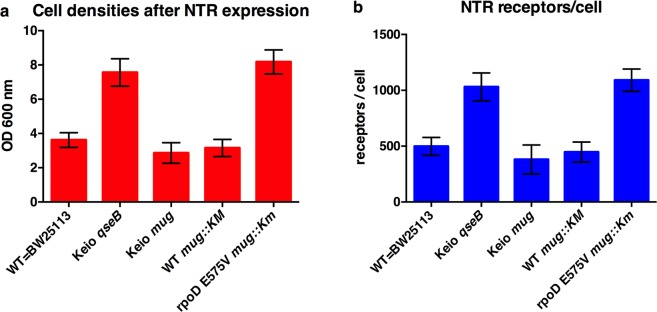
Expression of NTR1 in different strains. *E. coli* BW25113 (WT), Keio clones and the newly created strains were transformed with the plasmid pRG-NTR. (**a**) Growth was estimated with OD_600nm_ measurements after 20 hours of NTR1 expression at 20 °C. (**b**) Receptor expression levels were assessed by radioligand binding assays. Means and standard deviations from three independent experiments are shown.

Using these newly generated strains, we performed receptor expression experiments. Both the final cell density after expression and the functional receptor number per cell, estimated with RLBA, demonstrated that the improved phenotype displayed by the Keio clone *ΔqseB* is fully recapitulated and thus only due to this single point mutation E575V in *rpoD* (Fig. 3).

### Influence of medium

In all previous experiments, growth of the different bacterial strains was performed in the complex rich medium 2 × YT. In order to test the influence of the culture medium, we also investigated the EnPresso B system from BioSilta^23^. In this system glucose is enzymatically liberated from a polysaccharide substrate in a slow reaction. By this slow glucose-feeding, production of growth-inhibiting acids by the bacteria is reduced. Furthermore, the medium provides minerals, vitamins, and trace elements. As a result of these optimized medium conditions, higher cell densities can be reached with the EnPresso B system. Interestingly, we found that using the previously described protocol for receptor expression but using this optimized medium, the rpoD* strain can still reach double the cell density of the control strain when expressing wild-type NTR1 at 20 °C for 20 hours (Fig. S6a). Under these conditions, the newly developed rpoD* strain also produces more functional receptors per cell than the control strain (Fig. S6b).

Furthermore, we decided to also test the growth properties of the *E. coli* strains in minimal medium, as this would be important for future isotope labeling experiments. The selected Keio clones grew also to higher cell densities during expression of wild-type NTR1 at 20 °C (Fig. S7).

### Genetic background of other Keio clones

The *rpoD*-E575V mutation was found in the Keio clone *ΔqseB*, which had been enriched after iterative rounds of expression and selection using FACS from the Keio library transformed with an expression vector encoding wild-type NTR1. We verified by PCR of the genomic DNA and subsequent Sanger sequencing that this single point mutation was already present in the original Keio clone *ΔqseB* from the collection. However, we did not find the *rpoD*-E575V mutation in the other enriched clones, nor in wild-type BW25113.

We were thus interested to elucidate which mutations the other enriched clones from the Keio collection (*ΔhybD*, *ΔybaA*, and *ΔuxuB*) might carry, as they confer a similar phenotype. For this reason we also characterized them by whole genome sequencing. We found that all the three clones also have alterations exclusively in the sigma 70 factor gene *rpoD*: Keio strain *ΔhybD* has a deletion of 16 amino acids from position 172 to 187 (172|EDLAPTATHVGSELSQ); *ΔuxuB* carries the mutation I141S and *ΔybaA* the mutation D213Y (Fig. 2).

#### Other GPCRs

**Figure 4:**
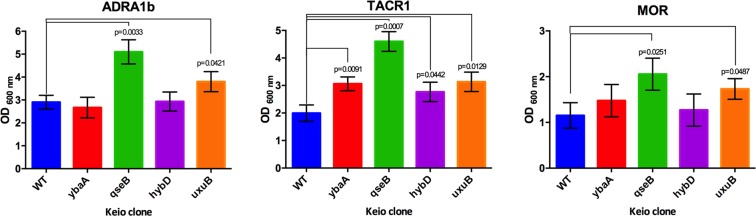
Cell growth after 20 h of GPCRs expression in different Keio clones. *E. coli* BW25113 (WT) and selected Keio clones were transformed with pRG plasmid derivatives encoding the wild-type version of ADRA1b, TACR1 and MOR GPCRs. Growth was estimated with OD_600nm_ measurement after 20 hours of GPCR expression at 20 °C. The x-axis label indicates the gene that is deleted in the respective Keio clone. Means and standard deviations from three independent experiments are shown. Statistical *p* values, as calculated by two-tailed paired *t* test, are indicated for strains with statistically significant increases in growth versus wild-type *E. coli* BW25113.

With the intention to test whether the observed phenotypes in the selected Keio clones are also obtained when other wild-type and hard to express GPCRs are expressed, we used the original Keio clones (*ΔhybD*, *ΔqseB*, *ΔybaA* and *ΔuxuB*) and transformed the strains with plasmid derivatives of pRG expressing alpha-1b adrenergic receptor (ADRA1b), tachykinin receptor (TACR), or μ-opioid receptor (MOR). Consistent with the previous results, these Keio collection clones also grow to significantly higher density than the wild-type control strain *E. coli* BW25113 when expressing TACR. For ADRA1b and TACR, Keio clones *ΔqseB* and to a lesser extent *ΔuxuB* can grow better during receptor expression (Fig. 4).

### Introducing the rpoD mutation into *E. coli* BL21

In order to prove that the point mutation in *rpoD* can also have an effect in a different genetic background, we generated a *rpoD*-E575V mutant of the BL21 Tuner strain which is frequently used for protein expression^24^. Indeed, the NTR1 expression experiments confirmed that the single point mutation E575V in *rpoD* also improved the growth and receptor yield in BL21 Tuner (Fig. S8), albeit to a somewhat smaller extent than in the BW25113 background. Comparable results were found when different GPCRs (ADRA1b, TACR and MOR) were analyzed (Fig. S9).

### Gene expression

The mutation E575V found in the sigma factor gene *rpoD* (Fig. 2) may have a role in the specific interaction between RNA polymerase and promoters during transcriptional activation. The receptor genes in the pRG vector are under control of the *lac* promoter, and thus regulated by sigma 70. Therefore, it is possible that this single point mutation modifies the mRNA levels in the bacteria, in particular the mRNA of the GPCR itself being expressed from the plasmid, but also that of other proteins.

#### qPCR

In order to test this hypothesis, we initially attempted to quantitate the relative mRNA amount of the NTR1 gene by using real-time qPCR. Different genes were tested as reference genes that had been suggested before as being constitutively expressed, for obtaining a relative quantification of NTR1: *ihfB*^25^; *cysG*, *hcaT*, and *idnT*^26^; and some genes suggested by the software Genevestigator from RefGenes^27^. Unfortunately, none of those fulfilled the requirements for a suitable reference gene under the tested conditions, as they were found themselves variable. Since the mutation in the sigma factor can have a broad and general impact on the expression of many genes, it may be unavoidable that it affects the potential reference genes as well.

#### Spike in

We thus used an exogenous RNA as a reference^28^, the Universal RNA Spike II (TATAA Biocenter). This allowed us also to test for inhibition of the enzymatic reactions inherent in amplification and estimate the RNA extraction yield. All samples taken from strains *E. coli* BW25113 wt (pRG-NTR) and *E. coli* BW25113 rpoD* (pRG-NTR) exhibited nearly identical Ct values (ΔCt < 0.2) for RNA Spike II.

Hence, using the RNA Spike II as a reference, we were able to determine that the expression of the NTR1 gene is down-regulated (log_2_ ratio = −2) in the rpoD* strain. Next, we also compared the expression of the NTR1 gene in the other Keio clones with mutations in *rpoD* gene (*ΔhybD*, *ΔybaA* and *ΔuxuB*). All the strains showed that NTR1 is down-regulated (log_2_ ratio near to −2) (Fig. S10).

#### RNA-seq

The mutation in *rpoD* may have, in addition to its direct effect on the GPCR gene, a more general effect on the expression of many genes. Therefore, we performed a whole transcriptome analysis (RNA-seq) of the *E. coli* strains under study, in order to find differentially expressed genes in a global and unbiased approach.

We prepared total RNA from the bacterial strains *E. coli* BW25113 wt and BW25113 rpoD*, both with and without the expression plasmid pRG-NTR, encoding the wt NTR1. Samples for RNA extraction were taken after 4 hours at 20 °C for NTR1 expression (when present). RNA-seq and the bioinformatics analysis were carried out as detailed in Supplementary Methods.

After quality control and processing, an average of 17 million reads and 94% of genomic features with reads above the threshold could be analyzed. To identify differentially expressed genes, we used a combined threshold of p-value (<0.01), fold-change >2 (log_2_ratio >1 or <−1) and at least 10 counts (for details, see Supplementary Methods)

A full set of gene expression data is shown in Table S1 and a summary of the number of up- and down-regulated genes found is shown in Table S2. A direct comparison of NTR1 expression in the wild-type strain versus the mutant strains shows that the level of receptor expression is lower in the mutant strain with a log_2_ ratio = −0.28, consistent with the qPCR data, albeit with a p-value of 0.068.

Table 1 shows a summary of the functional analysis of differentially expressed genes.

**Table 1:** Summary of gene enrichment analyses from RNA-seq data.

Strain	*E. coli* rpoD vs. *E. coli* wt	*E. coli* wt (NTR) vs. *E. coli* wt	*E. coli* rpoD (NTR) vs. *E. coli* wt (NTR)
UP	Glycerol degradation	Anaerobic respiration	Flagella - Sigma 28 regulon - FlhDC
	Plasma membrane proteins	Nitrate Reduction	CysB regulon
		Fermentation	Glycerol metabolism
		Glycolysis	Iron homeostasis (Sigma 19)
		Glycerol degradation	Sigma 70 regulon
		Electron Transfer	
		Transcription factor regulons: Crp, Cra, IHF, Nar, Fis, ArcA, Fnr	
		Sigma 38 regulon	
		Sigma 54 regulon	
		Sigma 32 regulon	
DOWN	Sigma 38 regulon	Flagella - Sigma 28 regulon - FlhDC	Anaerobic respiration
	Osmotic Stress	Plasma membrane proteins	Electron Transfer
	Starvation	Response to cold	Nitrate reduction
	Degradation of: amino acid, lipids, amines and carbohydrates	Protein secretion	Transcription factor regulons: Crp, Fnr, Cra, Fis, IHF, Nar, ArcA
		Arginine degradation	Sigma 38 regulon
		Lipopolysaccharide Metabolism Proteins	Sigma 32 regulon, protein folding chaperones

Compiled data of the enrichment analyses of the gene-expression from SmartTables and Omics Dashboard from BioCyc.org and the sigma factors dataset from RegulonDB analyzed with the Fisher exact statistics algorithm.

The *rpoD*-E575V mutation changes the expression levels of several genes in the *E. coli* BW25113 background, mainly by down-regulation (Fig. S11). Most genes under control of sigma factor 38, degradation pathways (amino acids, fatty acids and lipids, amines and carbohydrates), response to osmotic stress and starvation-related genes are down-regulated. On the other hand, pathways by which glycerol can be degraded to serve as a source of carbon and energy, as well as genes coding for plasma membrane components are up-regulated (Fig. S11).

When analyzing the effect of expressing NTR1 wt in the wt *E. coli* strain vs. the strain alone, we observed that receptor overexpression leads to an up-regulation of genes related to generation of energy, mainly anaerobic respiration, suggesting that receptor overexpression interferes with oxidative phosphorylation (Fig. S11). More genes controlled by sigma factor 32 (heat shock stress response), sigma 54 (nitrogen metabolism) and sigma 38 (general stress) are up-regulated than down-regulated, while sigma factor 28 dependent genes (motility and flagella) are largely down-regulated (Table S3). Also, transporter genes and those that are involved in protein type III secretion are significantly down-regulated, as well as plasma membrane components and lipopolysaccharide metabolism proteins (Fig. S11).

When genes are ordered by their log_2_ ratio according to the influence of expressing NTR1 in the *E. coli* wt, almost the mirror image is found when comparing *E. coli* wt and the *rpoD* mutant, both expressing NTR: the rpoD* mutation brings the physiology of *E. coli* back to where it was without expressing the GPCR NTR1 (Fig. S12). Most of the differentially expressed genes that are up-regulated when NTR1 is produced in the wt *E. coli* strain vs. the strain alone have lower expression values when the receptor is expressed in the mutant *rpoD* strain and *vice versa* (Table 1).

### Time course of NTR1 accumulation

**Figure 5:**
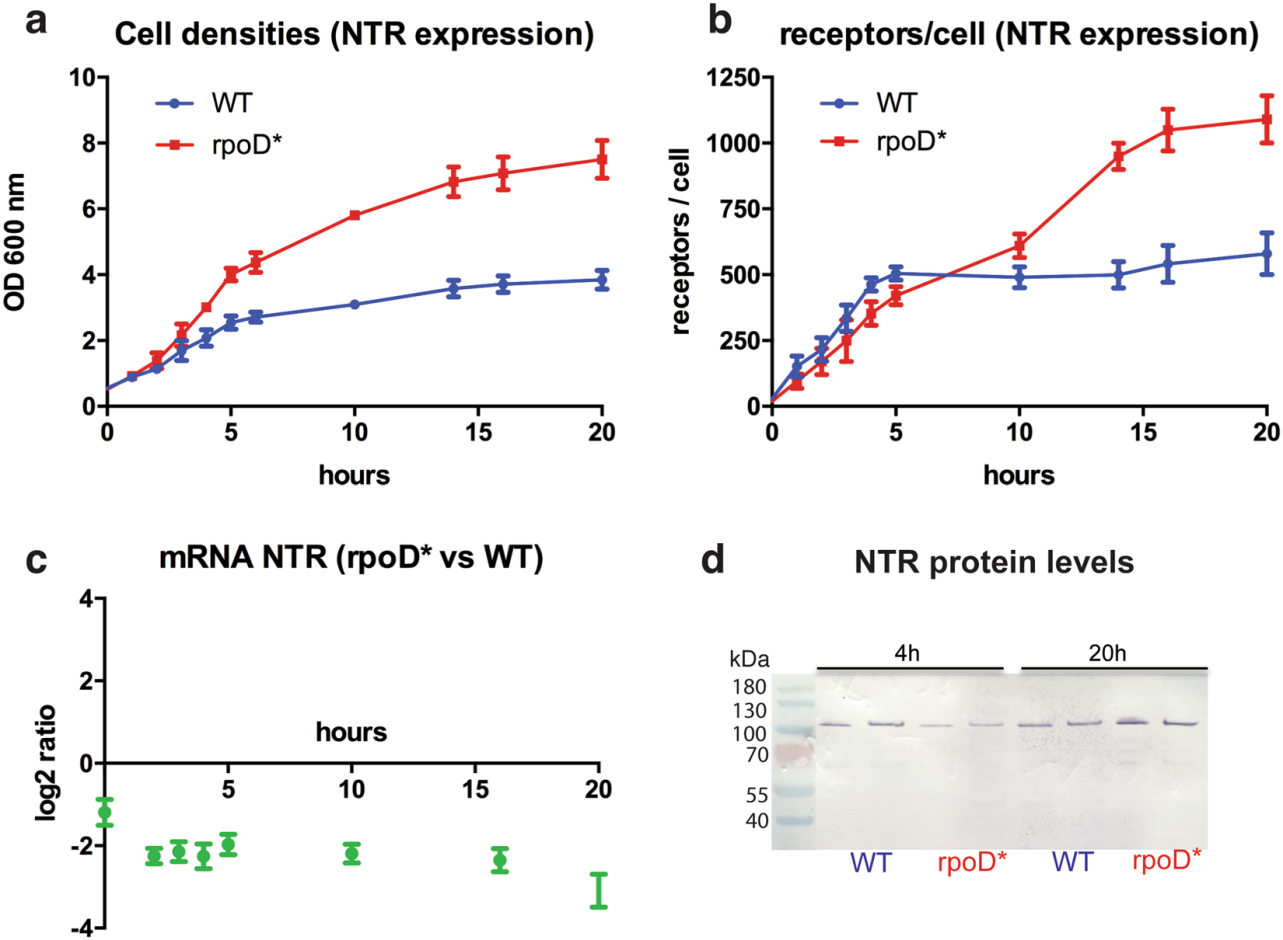
Kinetics of NTR1 expression. *E*. *coli* BW25113 (wt) and the *rpoD* E757V mutant strain were transformed with the plasmid pRG-NTR. NTR1 expression at 20 °C was performed for 20 hours. Samples were taken at different time points. (**a**) Growth was monitored with OD_600nm_ measurements. (**b**) Functional receptor levels were estimated by radioligand binding assays. (**c**) mRNA NTR1 levels were calculated by quantitative real-time PCR and expressed as log_2_ ratios between *rpoD* mutant vs. wt *E. coli* strain. (**d**) NTR1 protein levels were monitored with western blots using an anti-MBP antibody (in duplicates). The blot image has been cropped for conciseness. The band intensity data are not quantitative. The full-size blot is presented in Fig. S13.

In order to obtain a better understanding of NTR1 expression kinetics and whether the rpoD* mutation may influence it, cell samples of the wt and rpoD* mutant strains were taken as a function of time after induction of protein expression at 20 °C. At the same time, equivalent aliquots were used to estimate cell number (by measurement of optical density at 600 nm), functional receptors per cell (by RLBA), NTR1 gene expression (by qPCR) and total NTR1 protein amount by Western blot (Fig. 5).

The NTR1 gene expression rate is lower in the rpoD* strain over the whole time of receptor production. However, after approximately 4 hours of induction, the level of functional receptor in the wt strain seems to reach a plateau. In contrast, in the rpoD* mutant strain the level of functional NTR1 keeps increasing until 20 hours after protein induction. In parallel, cell growth in the wt strain has almost stopped after 5 hours, while the mutant strain continues to grow until the end of the experiment. Therefore, although the protein expression rate in the mutant *E. coli* strain is lower during the first hours, at later times it is still producing functional NTR1 protein, thereby reaching a higher level overall.

## Discussion

Overexpression of eukaryotic IMPs such as GPCRs in bacteria is usually toxic for the host. IMP toxicity is presumed to be due mainly to the overloading of the Sec translocon machinery^29^, which handles both the post-translational export of periplasmic proteins (via the SecB chaperone-targeting pathway) and the co-translational insertion (via the signal recognition particle (SRP)-targeting pathway) of most IMPs into the lipid bilayer membrane^30^. The translocation of proteins is hindered when the Sec translocon is saturated, possibly because of slow transport of heterologous membrane proteins and/or because of remaining interactions with the pore. Other toxic effects due to IMP expression include the accumulation of cytoplasmic protein aggregates and reduction of the levels of respiratory chain complexes in the bacterial membrane, which results in inefficient ATP production^31^.

Our aim was to investigate how *E. coli* cells could adapt to the stress of producing a toxic IMP and we wished to identify *E. coli* genes with an influence on GPCR expression. While our approach intended, in a first screen, to elucidate the effect of single gene deletions using the Keio collection (clean individual deletions of all nonessential genes of *E. coli*^18^), our study ultimately identified point mutations in the sigma factor 70, the *rpoD* gene, as responsible for a phenotype with significantly improved GPCR expression. The phenotypes of the enriched strains appeared indeed favorable for expressing wild-type GPCRs, essentially overcoming the stress imposed by the GPCR expression. From this point of view the selection has been very successful, but the mechanism was a different one than initially expected.

The point mutation E575V in the *rpoD* gene was found to be already existent in the original Keio clone *ΔqseB* from the collection. Other selected strains also turned out to have other mutations in the *rpoD* gene, with a less pronounced phenotype. The spontaneous mutation rate for *E. coli* has been estimated to be approximately 1 × 10^−3^ per genome per generation for cells cultured under standard laboratory conditions^32^. However, it should be noted that the spontaneous mutation rate is stress-dependent^33^. It is thus plausible that stress during the construction of the Keio strain collection could be the reason of the genetic instability of the *E. coli* BW25113 parent strain^34^, which has allowed the *rpoD* mutations to occur.

Bacteria control transcription initiation rates through regulation of sigma factor activity, a bacterial transcription initiation factor that enables specific binding of RNA polymerase to gene promoters. The sigma factor 70 (the *rpoD* gene product) is responsible for transcription of most genes expressed during the exponential cell growth^35^. Besides sigma 70, six other molecular species of alternative sigma factors in *E. coli* have been identified, present in the cell at different levels^35,36^. The accumulation of non-native or foreign structures in a membrane can induce stress responses and activate proteolytic systems of the overexpression host. Cells then produce stress-responsive proteins to alleviate the impact of the overexpressed protein and readjust their metabolism, predominantly via the heat-shock response of sigma 32 and sigma 24 pathways^37^, which compete with sigma 70 for the RNA polymerase core subunits. Mutations in sigma 70 may therefore not only affect the transcription initiation rates of corresponding promoters but also shift the balance between the other sigma factors.

The amino acid change Glu575 to Val in *rpoD*, leading to the most effective phenotype, lies in the first helix of the helix-turn-helix (HTH) motif in the carboxyl-terminal region of sigma 70 (Fig. 2). When we created the *rpoD*-E575V mutant in *E. coli* BL21(DE3) we found that the phenotype is also improved, albeit by a slightly lower amount. The reason for this smaller alteration may be that there is already a single amino acid difference in the *rpoD* wild-type gene between the BW25113 and BL21 strains in position 571, where BW25113 has E571, while BL21 and most of the *E. coli* strains carry H571.

Various mutations in the *rpoD* gene have been observed which affect transcription initiation that can be rationalized on the basis of the RNAP holoenzyme structure^38,39^. Jishage *et al*.^40^ reported that mutations such as *rpoD*-ΔDSA (536–538) and *rpoD*-Y571H have a reduced ability to bind to the RNA polymerase core and, for this reason, reduce their capability to compete for the core enzyme with the other sigma factors. These mutations have thus a similar consequence to that of Sigma 70 underproduction, or instead overproduction of the anti-sigma-70 factor Rsd^40^.

Several other examples of *rpoD* mutations have been described: some substitutions significantly decreased *ada*-dependent transcription, with rpoD E575V having the most severe effect, although the Glu-575 residue does not seem to be necessary for activator-independent transcription^41^; *rpoD-*E575A can reduce *uhpT* expression depending on the genetic background^42^; *rpoD*-R596, which increases expression of the *ara* regulon^43^; and *rpoD*-E575K which increases *lac* promoter activity^44^, while *pho* promoters are then less expressed^45^. Many of the *rpoD* mutations that were selected to alter the promoter recognition properties of RNA polymerase are in or near a conserved region of sigma 70 denoted as the helix-turn-helix motif (Fig. 2)^46^. Also, truncated *rpoD* mutants resulted in an increased ethanol tolerance in *E. coli*, due to a higher binding affinity of the truncated mutants to anti-sigma factors, relative to that of the full-length sigma 70 factor^47^. Additionally, in a study of laboratory evolution of pathway-engineered *E. coli* that under-produces the redox cofactor NADPH on glucose, the *rpoD*-D213Y discovered in our work was also found during their screening, although the authors did not provide details^48^. Collectively, these and our novel results underline that subtle changes in sigma 70, despite influencing a very general prokaryotic transcription factor, can change the relative individual expression levels of genes differentially.

We found that the GPCR gene is expressed at a lower rate in the rpoD* mutant *E. coli* strain when induced from the *lac* promoter, but that expression and cell growth both continue for a longer time, leading at the end to a 4-fold higher receptor yield per culture volume. This slower rate of biosynthesis seems to diminish the toxic effects of receptor expression. The simplest interpretation would be that all these mutations in the sigma 70 transcription factor reduce — directly or indirectly — the affinity of the RNA polymerase to the promoter, so that the transcriptional initiation frequency is lowered. This would lead to a slower but longer-lasting mRNA and GPCR production, less toxicity for the cell permitting longer cell growth, ultimately leading to higher GPCR expression levels.

This rpoD* effect is indeed seen for growth at 20 °C: GPCR expression at 37 °C causes a particularly high stress and is so toxic that even reducing the kinetics of expression is insufficient to make a significant difference in functional receptor expression. Interestingly, the bacteria with the rpoD* mutation can grow better and longer at 20 °C — independent of GPCR expression — even though the difference is not as pronounced as when overcoming stress through GPCR expression. This underlines the notion that the *rpoD* mutations must have additional beneficial effects for the cell at 20 °C by affecting other genes.

Whole transcriptome data of the *E. coli* strains were analyzed to study the effect of the stress-induced by GPCR overexpression and the effect of the selected *rpoD* mutations to dampen the effect on the cellular stress (Table S1). In either case, the observed up- or down-regulation of genes may be indirect, and not directly be under the control of the sigma factor under study.

Work from the de Gier group^31,49^ has shown the consequences of prokaryotic and eukaryotic membrane protein overexpression in *E. coli* at the protein level. Our transcriptome data described here for expression of NTR1 in the *E. coli* strain BW25113 have many correspondences with the proteomics data previously reported. We have likewise found up-regulated genes under the control of sigma 32 (heat shock response during log-phase growth), such as chaperones DnaK, IbpA, ClpB, GroELS, and the protease components HslVU; the activation of the Arc two-component system; the levels of enzymes involved in glycolysis (GapA, Pgk, Gpm, PykF) and in fermentation; as well as genes related to anaerobic respiration (Tables 1 and S1). In contrast, we find that sigma 24-dependent genes are both among the up- and down-regulated ones.

Although below the defined statistical cutoff, we detected increased levels of SecA, and found that some secreted proteins, like UgpB, TreA and ArgT, show a significantly decreased expression upon GPCR overexpression (Table S1). In addition, in our study the type III protein secretion system complex is down-regulated. All these observations suggest that the Sec translocon-mediated translocation of secretory proteins across the cytoplasmic membrane is also impaired upon NTR1 overexpression, potentially having an impact in the membrane protein composition, including the respiratory machinery. We likewise observed that the Arc two-component system response (redox sensor) and the phage shock protein A (PspA) response is activated upon receptor expression, consistent with cell envelope stress (Table S1).

Taken together, the consequences of overexpression of NTR1 in the *E. coli* strain BW25113 are similar to those of the overexpression of prokaryotic and other eukaryotic membrane proteins in other *E. coli* strains such as BL21(DE3)pLysS, C41(DE3) and C43(DE3) strains^49^.

In line with our findings, Link *et al*.^50^ demonstrated that overexpression of either FtsH or the cannabinoid receptor CB1, another GPCR, leads to induction of the heat-shock (sigma 32) stress responses. On the other hand, genes corresponding to the regulons of sigma 54 (nitrogen limitation), sigma 38 (general stress/ stationary phase) and sigma 28 (flagella synthesis) did not exhibit any noticeable variation upon expression of either CB1 or FtsH^50^.

The effect of the *rpoD*-E575V mutation when *E. coli* is grown at 20 °C (without NTR1 expression) can be rationalized in terms of what is known about bacterial adaptation to cold temperatures. *E. coli* exposed to a temperature decrease responds via cold-shock proteins termed Csps (mainly nucleic acid chaperones), increasing membrane fluidity (LpxP acyltransferase) and expression of stress-related genes controlled by sigma factor 38 (RpoS)^51^. White-Ziegler *et al*.^52^ reported that almost half of the genes up-regulated at 23 °C compared with those at 37 °C were controlled by sigma factor^38^. Interestingly, our transcriptome analysis indicates that most of those genes related to cold shock response are down-regulated in the *rpoD* mutant strain compared to the wt *E. coli* strain upon growth at 20 °C (Tables 1 and S1). This can be interpreted as the *rpoD* mutation allowing better adaptation to growth at lowers temperatures, and that these additional cold shock responses are less essential.

It thus appears that the selection of a mutation in *rpoD* may have two independent beneficial effects; first, a decrease in expression rate of the toxic GPCR itself and second, a “renormalization” of the genes from the stress phenotype to one of normally growing *E. coli* (Fig. S11). While part of this effect can be indirect, i.e., by decreasing the stress in a cell that produces the receptor more slowly, we would like to reiterate that the rpoD* strain also has improved growth properties at low temperature in the absence of GPCR expression.

It is probably for this dual role why the *rpoD* mutations appear to have an additional benefit over the straightforward lowering of expression rate by low inducer level, the use of different (weaker) promoters or the use of low copy number plasmids.

A general picture thus emerges in that the NTR1 expression rate from the plasmid pRG-NRT1 in the rpoD* mutant strain is always lower than in the wt. However, the level of functional receptor and the cell growth stopped after 4 hours of production in the wt strain. In contrast, the mutant strain continued to grow without problems and kept on synthetizing functional NTR1 for at least 20 hours after induction. Therefore, the final functional GPCR yields are increased in the rpoD* mutant strain, thanks to the reduction in the transcription rate. *In addition*, the cell altered protein expression patterns, as indicated in Table 1 and in more detail in Supplemental Table S1, to further alleviate the stress response. It is notable that all clones identified by selection for functional GPCR expression have mutations in *rpoD*, and among this strain collection, no other gene was found to improve GPCR expression.

Different genetic approaches to randomly mutate *E. coli* in order to obtain strains with improved membrane protein overexpression characteristics have also ultimately led to a lowering of expression rates^53–55^. *E. coli* BL21(DE3) harbors a phage λDE3 lysogen that carries the gene for T7 RNA polymerase under control of the *lacUV5* promoter. When membrane proteins were expressed in this system, they frequently showed signs of strong toxicity and most of the cells died. Selecting for survivors, the strains C41(DE3) and C43(DE3) were found^53^, which later on were identified to have accumulated “weakening” mutations in the *lacUV5* promoter, leading to reduced synthesis of the T7 polymerase upon induction with IPTG^54^. Afterward, whole genome sequencing identified mutations in *yehU* (encoding a putative peptide transporter), *rbsD::IS150* (the mutation is enabling efficient growth on ribose), and *dcuS_ps* (the mutation converts the pseudogene into an enzyme allowing efficient utilization of C4-dicarboxylates)*;* however these additional mutations appear to have been the prerequisite for the *lacUV5* promoter being able to mutate, rather than being directly linked to improved IMP overexpression^56^. In addition, mutant V192F of the lac repressor gene *lacI* binds more tightly to the lac-operator, and thus being a stronger repressor, improving overexpression to certain membrane proteins in C41(DE3)^57^. In a yet another approach, the strain Lemo(DE3) was constructed, where the gene encoding the T7 lysozyme, an inhibitor of T7-RNA polymerase, was placed under the control of the well titratable rhamnose promoter^58^. As additional example, another *E. coli* BL21(DE3) mutant was isolated, that contains point mutations in the gene encoding the T7 RNAP, lowering the affinity of the polymerase for the T7 promoter^59^. Both cases reduce T7 RNA polymerase mediated protein synthesis.

In the previous examples, the slower transcription rates of the target membrane protein comes from the decreased intracellular levels of the highly processive T7 RNA polymerase, which is thus reducing the amount of mRNA-template available for translation^49^. Therefore, those strain selections have addressed to some extent the same principle as in the present study, namely the reduction of synthesis rate (production per time unit) as one of the stress factors for IMP production. However, the *rpoD* mutations uncovered in the present work additionally alter the relative expression level of a number of genes that are related with stress as well as growth at low temperature, since the *rpoD* * strains were found to grow for longer times under these conditions. Coexpression of some known bacterial components^60,61^ has also been investigated, and a transposon insertion library showed that *deletion* of a chaperone resulted in increased expression (albeit in an inactive form) of one GPCR^62^. Nonetheless, it seems that the dual action of the rpoD* mutation in altering the level of host genes, in conjunction with a slower expression rate of the GPCR, leads to the beneficial effects on GPCR expression and growth duration found in the present work.

Another example of an *E. coli* strain with improved membrane protein overexpression characteristics was isolated by Massey-Gendel and colleagues^55^, showing a reduced copy number of the overexpression vectors. In yet a different approach, a single nucleotide transversion in one of the CRP interaction domains of the pBAD arabinose promoter was isolated that reduces the transcription rates by about 70% and can improve the yields of some membrane proteins^63^.

In all these examples the unifying feature is the lowered expression rate that improves membrane protein overexpression yields. In contrast, with the *rpoD* mutations found here, we achieved an additional fine tuning which was not entirely reached with the more traditional procedures of lowering expression only, being by promoter strength or plasmid copy number. Indeed, for the GPCRs studied here, it was found necessary, but not sufficient to decrease the translation rate by lowering the culture temperature or inducer concentration, or by choosing a weaker promoter. The global *rpoD* effect resulted in additional benefits due to the longer growth and thus expression duration of the bacterial cells.

## Conclusions

While we initially started off from a single-gene deletion strategy in searching for strains with improved functional GPCR expression properties, we found no single gene with these properties. Instead, our selection uncovered subtle mutations in *rpoD* as the recurring motif in all mutant strains with the desired phenotype. The altered sigma factor 70 has an effect on the receptor expression itself, in that the rate of biosynthesis at 20 °C is lowered, but it continues for much longer, and cell growth is also continuing in parallel, resulting in a 4-fold higher final functional GPCR yield in the mutants rpoD* strain. Importantly, the *rpoD* mutations also affect the strain itself, as it grows to higher densities at 20 °C, even in the absence of GPCR expression, and this effect is more pronounced when it overcomes the growth inhibition due to stress in the presence of a GPCR. We found that the mutated sigma 70 alters the relative expression level of many genes, and thus also has a general effect on alleviating stress from the overexpression of the GPCR at several levels, contributing to the desirable phenotype.

These findings can now be exploited in further strain engineering, as optimization of the expression of other membrane protein families or even other toxic soluble proteins may need a different optimized rpoD mutant. The development of expression strains with superior capability for heterologous functional membrane protein production may be particularly useful for isotope-labeled proteins as used in NMR for which *E. coli* is by far the most convenient expression system. Transcriptional factor fine-tuning may provide a different way of coordinate and well-adjust the biogenesis of membrane proteins.

## Materials and Methods

### Materials

Unless otherwise noted, chemicals were of the highest quality obtainable and purchased from Sigma or AppliChem. Enzymes for molecular biology were from New England Biolabs or Fermentas. All [^3^H]-labeled ligands were obtained from Perkin Elmer: [3,11-tyrosyl-3,5-^3^H(N)]-neurotensin (NT); [7-methoxy-^3^H]-prazosin; 9-sar,11-met(O2)[2-prolyl-3,4-^3^H]-Substance-P and [tyrosyl-3,5-^3^H(N)]-[D-Ala2,*N*-MePhe4,Gly-ol]-enkephalin (DAMGO) were purchased from Perkin-Elmer. All unlabeled ligands were purchased from AnaSpec. The fluorescent ligand HL647-NT(8–13) was obtained by custom synthesis from Anawa. (NT(8–13) was labeled at the N-terminus with the fluorescent dye HiLyte Fluor 647 from AnaSpec.

### Bacterial cultures

Bacterial cultures were grown in 2 × YT rich medium or, where indicated, in minimal medium: M9 salts, 1% (w/v) glucose, 0.5 mM thiamine hydrochloride, 10 mM MgSO_4_, 0.1 mM CaCl_2_ and a trace metal solution (Fe, Al, Mn, Co, Zn, Cu, and H_3_BO_3_).

### Keio collection

The Keio collection is a systematically constructed library of *E. coli* mutants with 3985 precisely defined single-gene deletions representing all non-essential genes^18^. Each deleted target gene was replaced by a kanamycin resistance cassette^19^. The background genotype of the collection is the *E. coli* strain BW25113 [*Δ(araD-araB)567 ΔlacZ4787(::rrnB-3)−λ*^*−*^ *rph-1 Δ(rhaD-rhaB)568 hsdR514*]. The collection was obtained from the Nara Institute of Science and Technology, Keio University, Japan, and Purdue Research Foundation.

### Protein expression

All GPCR variants were expressed as fusion proteins containing maltose binding protein (MBP) at the N-terminus and thioredoxin A (TrxA) at the C-terminus in a pBR322-derived vector denoted as pRG, as previously described^8,10^. Briefly, receptors were expressed in 2 × YT medium supplemented with 0.2% (w/v) glucose and 100 μg/ml ampicillin. The cells were grown at 37 °C until the culture reached OD_600_ = 0.5. At this cell density, protein expression was induced by addition of 250 μM IPTG and the cells were cultivated at 20 °C for 20 h.

### FACS-based selections

FACS-based selections were performed as previously described^10,11^. Briefly, cells were permeabilized after receptor expression by washing with TKCl buffer (50 mM Tris-HCl, 150 mM KCl, pH 7.4 at 4 °C). After permeabilization, binding of fluorescent ligand was performed in TKCl buffer supplemented with 80 nM HL647-NT (8–13) at 4 °C without exposure to light for 2 h. For measurement of the non-specific signal, competitive ligand binding was performed with the TKCl buffer containing, in addition to the fluorescent ligand, also 80 µM unlabeled NT(8–13). After ligand binding, cells were washed and resuspended in TKCL buffer and sorting was done on a BD FACS Aria I cell sorter (BD Biosciences). Sorted cells were collected in 2 × YT medium supplemented with 1% (w/v) glucose and 100 μg/ml ampicillin and grown overnight at 37 °C.

### Inverse PCR

Genomic DNA (gDNA) from each clone to be analyzed was extracted from approximately 5 × 10^9^ *E. coli* cells with the GenElute™ Bacterial Genomic DNA Kit (Sigma-Aldrich, Cat. No. NA2110). 3 μg of genomic DNA were digested with 100 units of the restriction enzyme MseI (New England Biolabs) at 37 °C for 5 h. Due to its four-nucleotide recognition site, MseI cuts the genomic DNA frequently. However, there is no MseI site present within the kanamycin cassette, which replaces the deleted genes in the clones of the Keio collection. The digested DNA was purified with the QIAquick PCR purification kit (Qiagen, Cat. No. 28104). In order to promote intramolecular ligation of the genomic DNA fragments to give circular DNA, the digested DNA was diluted. For this purpose, approximately 75 ng of each digested DNA was used for a 100-μl ligation reaction. Ligation was performed with T4 DNA ligase (Thermo Fisher Scientific) at 16 °C for 18 h. Subsequently, the ligated DNA fragments were purified with the QIAquick PCR purification kit. The DNA fragment containing the kanamycin resistance cassette and parts of the adjacent DNA regions was then amplified in a PCR reaction using Phusion High-Fidelity DNA polymerase (New England Biolabs) and outward primers annealing to the kanamycin resistance cassette (forward primer: 5′-TGCTTTACGGTATCGCCGCTCC-3′; reverse primer: 5′-ACATTCATCCGGGGTCAGCACC-3′). The PCR product was then purified with the QIAquick PCR purification kit and subsequently sent to sequencing, allowing the identification of the deleted gene by analysis of the DNA sequence adjacent to the kanamycin resistance cassette.

### Radioligand-binding assays

Whole-cell radioligand-binding assays (RLBAs) *in E. coli* were performed as previously described^12^. The [^3^ H]-labeled ligands (NT, Prazosin, Substance-P and DAMGO) were used at 20 nM and competed with 20 μM unlabeled ligand to determine the non-specific signal.

### Chromosomal gene deletions

The procedure to delete genes in the *E. coli* BW25513 chromosome was adapted from Datsenko and Wanner^19^ and Baba *et al*.^18^. To delete a gene in *E. coli* BL21, the Quick and Easy *E. coli* gene deletion kit using Red/ET recombination was used (Gene Bridges, Cat. No. K006).

### Western blots

In order to load the same number of cells of each strain, *E. coli* whole cell protein extracts were obtained from 1 × 10^8^ cells, as estimated by optical densities measured at 600 nm. These normalized numbers of cells were resuspended in 50 µl reducing NuPAGE LDS sample buffer (Life Technologies, Cat. No. NP0008). Samples were incubated at 25 °C for 15 min with mixing, centrifuged, and 10 µL of each sample were run at 100 V for 3 h on a NuPAGE 4–12% Bis-Tris protein gel (Life Technologies, Cat. No. NP0335) in NuPAGE MES SDS running buffer (Life Technologies, Cat. No. NP0002) supplemented with NuPAGE sample reducing agent (Life Technologies, Cat. No. NP0004) and NuPAGE antioxidant (Life Technologies, Cat. No. NP0005). Proteins were transferred to Immobilon PVDF-FL membranes (Merck Millipore, Cat. No. PFL00010) activated in methanol by using a Trans-Blot® SD Semi-Dry Transfer Cell (Bio-Rad) at 10 V for 1 h. Blocking of membranes was performed in 1 × casein blocking buffer (Sigma-Aldrich, Cat. No. C7594) in PBS at RT for 20 min. Antibody binding was performed in 1 × casein blocking buffer in PBST (PBS, 0.1% (v/v) Tween-20) at RT for 1 h, and PBST was used for all membrane washing steps. Primary mouse anti-MBP antibody was used at a dilution of 1:4,000 and the secondary antibody alkaline phosphatase anti mouse IgG at a dilution of 1:10,000. Detection was achieved with NBT-BCIP (ThermoFischer, Cat. No. 34042) in detection buffer (100 mM Tris-HCl, 100 mM NaCl, 5 mM MgCl_2_, pH 9.5 at 25 °C).

### Whole genome sequencing

Sequencing of whole genome was carried out both with PacBio RSII and Illumina MiSeq. The methods are described in detail in the Supplementary Methods.

### Site-directed mutagenesis in the *E. coli* genome

This was carried combining the method of Datsenko and Wanner for gene deletions^19^ with Splicing by Overlap Extension^64^, and is described in detail in the Supplementary Methods.

### RNA-sequencing

Sample preparation was done with the TruSeq RNA Sample Prep kit v2 with ribosomal-RNA-depleted total RNA samples. RNA sequencing was done with the TruSeq SR Cluster Kit v4-cBot-HS. Details on sample preparation and methods are explained in the Supplementary Methods.

### Bioinformatics

The software packages and methods used are described in detail in the Supplementary Methods.

### Quantitative real time PCR

Quantitative PCR was performed following the MIQE guidelines and using a Mx3005P qPCR System. Sample preparation details and evaluation are explained in the Supplementary Methods.

## Supplementary information

Supplementary Information

Supplementary Table 1

## Data Availability

All data generated and analyzed during this study are included in this published article (and its Supplementary Information files). The RNA sequencing reads and raw counts have been deposited in Gene Expression Omnibus of NCBI under accession number GSE109819.

## References

[1] Almén M, Nordström KJ, Fredriksson R, Schiöth HB (2009). Mapping the human membrane proteome: a majority of the human membrane proteins can be classified according to function and evolutionary origin. BMC Biol..

[2] Carpenter EP, Beis K, Cameron AD, Iwata S (2008). Overcoming the challenges of membrane protein crystallography. Curr. Opin. Struct. Biol..

[3] Cherezov V, Abola E, Stevens RC (2010). Recent progress in the structure determination of GPCRs, a membrane protein family with high potential as pharmaceutical targets. Methods Mol. Biol..

[4] Wagner S, Bader ML, Drew D, de Gier J-W (2006). Rationalizing membrane protein overexpression. Trends Biotechnol..

[5] Schlegel S (2010). Revolutionizing membrane protein overexpression in bacteria. Microb. Biotechnol..

[6] Wang D-N (2003). Practical aspects of overexpressing bacterial secondary membrane transporters for structural studies. Biochim. Biophys. Acta (BBA) - Biomembranes.

[7] Gardner KH, Kay LE (1998). The use of 2H, 13C, 15N multidimensional NMR to study the structure and dynamics of proteins. Annu. Rev. Biophys. Biomol. Struct..

[8] Tucker J, Grisshammer R (1996). Purification of a rat neurotensin receptor expressed in Escherichia coli. Biochem. J..

[9] Martinez Molina D (2008). Engineering membrane protein overproduction in Escherichia coli. Protein Sci..

[10] Sarkar CA (2008). Directed evolution of a G protein-coupled receptor for expression, stability, and binding selectivity. Proc. Natl. Acad. Sci. USA.

[11] Dodevski I, Plückthun A (2011). Evolution of three human GPCRs for higher expression and stability. J. Mol. Biol..

[12] Schlinkmann KM (2012). Maximizing detergent stability and functional expression of a GPCR by exhaustive recombination and evolution. J. Mol. Biol..

[13] Schlinkmann KM (2012). Critical features for biosynthesis, stability, and functionality of a G protein-coupled receptor uncovered by all-versus-all mutations. Proc. Natl. Acad. Sci. USA.

[14] Schütz M (2016). Directed evolution of G protein-coupled receptors in yeast for higher functional production in eukaryotic expression hosts. Sci. Rep..

[15] Freigassner M, Pichler H, Glieder A (2009). Tuning microbial hosts for membrane protein production. Microb. Cell Fact..

[16] Makrides SC (1996). Strategies for achieving high-level expression of genes in Escherichia coli. Microbiol. Rev..

[17] Grisshammer R (2006). Understanding recombinant expression of membrane proteins. Curr. Opin. Biotechnol..

[18] Baba T (2006). Construction of Escherichia coli K-12 in-frame, single-gene knockout mutants: the Keio collection. Mol. Syst. Biol..

[19] Datsenko KA, Wanner BL (2000). One-step inactivation of chromosomal genes in Escherichia coli K-12 using PCR products. Proc. Natl. Acad. Sci. USA.

[20] Grenier F, Matteau D, Baby V, Rodrigue S (2014). Complete genome sequence of Escherichia coli BW25113. Genome Announc..

[21] Gruber TM, Gross CA (2003). Multiple sigma subunits and the partitioning of bacterial transcription space. Annu. Rev. Microbiol..

[22] Lonetto MAEA, Gribskov M, Gross CA (1992). The sigma 70 family: sequence conservation and evolutionary relationships. J. Bacteriol..

[23] Ukkonen K, Vasala A, Ojamo H, Neubauer P (2011). High-yield production of biologically active recombinant protein in shake flask culture by combination of enzyme-based glucose delivery and increased oxygen transfer. Microb. Cell Fact..

[24] Egloff P (2014). Structure of signaling-competent neurotensin receptor 1 obtained by directed evolution in Escherichia coli. Proc. Natl. Acad. Sci. USA.

[25] Aguilar C (2012). Genetic changes during a laboratory adaptive evolution process that allowed fast growth in glucose to an Escherichia coli strain lacking the major glucose transport system. BMC Genomics.

[26] Zhou K (2011). Novel reference genes for quantifying transcriptional responses of Escherichia coli to protein overexpression by quantitative PCR. BMC Mol. Biol..

[27] Hruz T (2011). RefGenes: identification of reliable and condition specific reference genes for RT-qPCR data normalization. BMC Genomics.

[28] Devonshire AS, Elaswarapu R, Foy CA (2010). Evaluation of external RNA controls for the standardisation of gene expression biomarker measurements. BMC Genomics.

[29] Valent QA (1998). The Escherichia coli SRP and SecB targeting pathways converge at the translocon. EMBO J..

[30] Luirink J, Yu Z, Wagner S, de Gier J-W (2012). Biogenesis of inner membrane proteins in Escherichia coli. Biochim. Biophys. Acta (BBA) - Bioenergetics.

[31] Wagner S (2007). Consequences of membrane protein overexpression in Escherichia coli. Mol. Cell Proteomics.

[32] Lee H, Popodi E, Tang H, Foster PL (2012). Rate and molecular spectrum of spontaneous mutations in the bacterium Escherichia coli as determined by whole-genome sequencing. Proc. Natl. Acad. Sci. USA.

[33] Maharjan R, Ferenci T (2015). Mutational signatures indicative of environmental stress in bacteria. Mol. Biol. Evol..

[34] Zhou J, Rudd KE (2013). EcoGene 3.0. Nucleic Acids Res..

[35] Jishage M, Iwata A, Ueda S, Ishihama A (1996). Regulation of RNA polymerase sigma subunit synthesis in Escherichia coli: intracellular levels of four species of sigma subunit under various growth conditions. J. Bacteriol..

[36] Maeda H, Fujita N, Ishihama A (2000). Competition among seven Escherichia coli σ subunits: relative binding affinities to the core RNA polymerase. Nucleic Acids Res..

[37] Chou CP (2007). Engineering cell physiology to enhance recombinant protein production in Escherichia coli. Appl. Microbiol. Biotechnol..

[38] Murakami KS (2013). X-ray Crystal structure of Escherichia coli RNA polymerase 70 holoenzyme. J. Biol. Chem..

[39] Zuo Y, Steitz TA (2015). Crystal structures of the E. coli transcription initiation complexes with a complete bubble. Molecular Cell.

[40] Jishage M, Kvint K, Shingler V, Nyström T (2002). Regulation of sigma factor competition by the alarmone ppGpp. Genes & Development.

[41] Landini P, Bown JA, Volkert MR, Busby SJW (1998). Ada protein-RNA polymerase σ subunit interaction and α subunit-promoter DNA interaction are necessary at different steps in transcription initiation at the Escherichia coli ada and aidB Promoters. J. Biol. Chem..

[42] Olekhnovich IN, Kadner RJ (1999). RNA polymerase α and σ70 subunits participate in transcription of the Escherichia coli uhpT promoter. J. Bacteriol..

[43] Hu JC, Gross CA (1988). Mutations in rpoD that increase expression of genes in the mal regulon of Escherichia coli K-12. J. Mol. Biol..

[44] Siegele DA, Hu JC, Gross CA (1988). Mutations in rpoD, the gene encoding the sigma 70 subunit of Escherichia coli RNA polymerase, that increase expression of the lac operon in the absence of CAP-cAMP. J. Mol. Biol..

[45] Makino K, Amemura M, Kim SK, Nakata A, Shinagawa H (1993). Role of the sigma 70 subunit of RNA polymerase in transcriptional activation by activator protein PhoB in Escherichia coli. Genes & Development.

[46] Siegele DA, Hu JC, Walter WA, Gross CA (1989). Altered promoter recognition by mutant forms of the sigma 70 subunit of Escherichia coli RNA polymerase. J. Mol. Biol..

[47] Tan F-R (2015). Improving furfural tolerance of Zymomonas mobilis by rewiring a sigma factor RpoD protein. Appl. Microbiol. Biotechnol..

[48] Chou H-H, Marx CJ, Sauer U (2015). Transhydrogenase promotes the robustness and evolvability of E. coli deficient in NADPH production. PLoS Genet..

[49] Klepsch MM, Persson JO, de Gier J-WL (2011). Consequences of the overexpression of a eukaryotic membrane protein, the human KDEL receptor, in Escherichia coli. J. Mol. Biol..

[50] Xu LY, Link AJ (2009). Stress responses to heterologous membrane protein expression in Escherichia coli. Biotechnol. Lett..

[51] Barria C, Malecki M, Arraiano CM (2013). Bacterial adaptation to cold. Microbiology.

[52] White-Ziegler CA (2008). Low temperature (23 degrees C) increases expression of biofilm-, cold-shock- and RpoS-dependent genes in Escherichia coli K-12. Microbiology.

[53] Miroux B, Walker JE (1996). Over-production of proteins in Escherichia coli: mutant hosts that allow synthesis of some membrane proteins and globular proteins at high levels. J. Mol. Biol ..

[54] Wagner S (2008). Tuning Escherichia coli for membrane protein overexpression. Proc. Natl. Acad. Sci. USA.

[55] Massey-Gendel E (2009). Genetic selection system for improving recombinant membrane protein expression in E. coli. Protein Sci..

[56] Schlegel S, Genevaux P, de Gier J-W (2015). De-convoluting the genetic adaptations of E. coli C41(DE3) in real time reveals how alleviating protein production stress improves yields. Cell Rep..

[57] Kwon S-K, Kim SK, Lee D-H, Kim JF (2015). Comparative genomics and experimental evolution of Escherichia coli BL21(DE3) strains reveal the landscape of toxicity escape from membrane protein overproduction. Sci. Rep..

[58] Schlegel S (2012). Optimizing membrane protein overexpression in the Escherichia coli strain Lemo21(DE3). J. Mol. Biol..

[59] Baumgarten T (2017). Isolation and characterization of the E. coli membrane protein production strain Mutant56(DE3). Sci. Rep..

[60] Link AJ, Skretas G, Strauch E-M, Chari NS, Georgiou G (2008). Efficient production of membrane-integrated and detergent-soluble G protein-coupled receptors in Escherichia coli. Protein Sci..

[61] Skretas G, Georgiou G (2010). Simple genetic selection protocol for isolation of overexpressed genes that enhance accumulation of membrane-integrated human g protein-coupled receptors in Escherichia coli. Appl. Environ. Microbiol..

[62] Skretas G, Georgiou G (2009). Genetic analysis of G protein-coupled receptor expression in Escherichia coli: Inhibitory role of DnaJ on the membrane integration of the human central cannabinoid receptor. Biotechnol. Bioeng..

[63] Nannenga BL, Baneyx F (2011). Enhanced expression of membrane proteins in E. coli with a P(BAD) promoter mutant: synergies with chaperone pathway engineering strategies. Microb. Cell Fact..

[64] Horton RM (1993). Gene splicing by overlap extension. Meth. Enzymol..

[65] Paget M (2015). Bacterial sigma factors and anti-sigma factors: structure, function and distribution. Biomolecules.

