# Supplementary material for: Mutations in sigma 70 transcription factor improves expression of functional eukaryotic membrane proteins in Escherichia coli
Source: Sci Rep. 2019 Feb 21;9:2483. doi: 10.1038/s41598-019-39492-9 (PMC6384906; doi:10.1038/s41598-019-39492-9)
Supplement: Supplementary file 1 — Supplementary Information [file 41598_2019_39492_MOESM1_ESM.pdf]

Title

Mutations in sigma 70 transcription factor improves expression of functional eukaryotic membrane proteins in *Escherichia coli*

Authors

Pablo Emiliano Tomatis[1], Marco Schütz[2], Elina Umudumov[3] and Andreas Plückthun*

Supplementary Methods

Whole genome sequencing

The genomic DNA of the clone to be sequenced was extracted from approximately 1×10^9 *E. coli* cells with the GenElute™ Bacterial Genomic DNA Kit (Sigma, Cat. No. NA2110) and quantified with the Quant-iT™ PicoGreen ds DNA kit (Invitrogen, Cat. No. P7589). Prior to sequencing, the quality of the isolated genomic DNA was checked with a Bioanalyzer 2100 instrument (Agilent Technologies).

The genomes were sequenced using either 4 single-molecule real-time sequencing (SMRT) cells on a PacBio RS II or an Illumina MiSeq instrument (NextXT library kit), both at the Functional Genomics Center Zurich. The concentration of the input DNA was determined by using the Qubit Fluorometer dsDNA Broad Range assay (Life Technologies, Cat. No. Q32850).

PacBio RS II. The DNA sequence of the whole genome of Keio clone *Δ qseB* and the wild-type strain BW25113 were obtained using PacBio RS with SMRT cells to get long pair-end reads and thus to be able to also detect large rearrangements.

The SMRT bell was produced using the DNA Template Prep Kit 2.0 (Pacific Biosciences, Cat. No. 001-540-835) according to the 3-kb or 10-kb template preparation and sequencing protocol provided by Pacific Biosciences. 10 µg of genomic DNA were mechanically sheared to an average size distribution of 10 kb, using a Covaris gTube (Kbiosciences Cat. No 520079). A Bioanalyzer 2100 12K DNA Chip assay (Agilent Technologies, Cat. No. 5067-1508) was used to assess the fragment size distribution. 5 µg of sheared genomic DNA were incubated with polishing enzymes to repair damages at the ends of the DNA fragments. A blunt-end ligation reaction followed by exonuclease treatment was performed to create the SMRT bell template. The quality of the library was inspected with the Agilent Bioanalyzer 12Kb DNA Chip and the Qubit Fluorimeter. A ready-to-sequence SMRT bell-polymerase complex was created using the P4 DNA/Polymerase binding kit 2.0 according to the manufacturer's instructions (Pacific Biosciences, Cat. No. 100-236-500).

The Pacific Biosciences RS2 instrument was programmed to load and sequence the sample on 4 SMRT cells v3.0 per sample (Pacific Biosciences, Cat. No. 100-171-800), recording 1 movie of 120 minutes each per SMRT cell. A MagBead loading (Pacific Biosciences, Cat. No 100-133-600) method was chosen in order to improve the enrichment of the longer fragments. After the run, a sequencing report was generated for every cell via the SMRT portal, in order to assess the adapter dimer contamination, the sample loading efficiency, the obtained average read-length and the number of filtered sub-reads.

A total of 71682/71237 reads with a mean length of 3332/3621 bp were assembled with a 20/30 fold coverage into 1/4 contigs for the wild type BW25113 strain/ Keio clone *Δ qseB*, respectively. The genomes were compared and the replacement of the *qseB* gene by the kanamycin resistance cassette in the Keio clone was confirmed.

Illumina MiSeq. As Nextera XT requires a maximum of 1 ng of total genomic DNA in 5 µl of starting volume, each sample was diluted to a concentration of 0.2 ng/µl genomic DNA as input dsDNA. The library preparation with individual library barcoding and normalization of the respective libraries was performed using the Nextera XT kit (Illumina, Cat. No. FC-131-1096) according to the manufacturer's protocol. Nextera XT

libraries were quantified using Qubit and the size profile was analyzed on the 2200 TapeStation (Agilent). The libraries were pooled together and diluted to 4 nM. The library pool was denatured and further diluted prior to loading on a MiSeq paired-end 500 cycle (v2) sequencing run. We thus obtained a pattern of sequencing 2x250bp and a minimum genome coverage of 25x on average.

Site-directed mutagenesis in the *E. coli* genome

We developed a method for making site-directed mutagenesis in the *E. coli* genome. For this purpose, we use the methodology named Splicing by Overlap Extension [1] to create a DNA fusion between the kanamycin resistance cassette targeted to the non-essential *mug* gene (downstream of *rpoD*) and the last 350 bp of the *rpoD* gene. We use a DNA fragment containing the required mutation (rpoD-E575V) and also in parallel the wild-type *rpoD* sequence. Next, we followed the Datsenko method for gene deletions [2], using the fused DNA fragment created as input, and we then used the kanamycin resistance for selection of the new *E. coli* strains that only differ by the desired point mutation.

To be able to use this method with the *E. coli* BL21 strain, we needed to integrate the *recA* gene from *E. coli* to the lambda Red recombinase system, as this gene is deleted in the BL21 strain. This was done by using the Red/ET recombination kit from Gene Bridges® [3], instead of the Datsenko and Wanner plasmids.

RNA-sequencing

Total RNA was extracted from approximately 5 × 10^8 *E. coli* cells using the RNeasy Mini kit (Qiagen, Cat. No. 74104). Briefly, bacterial cell cultures were directly mixed with twice the volume of RNA-protect Bacteria Reagent (Qiagen, Cat. No. 76506) and the recommended protocol of lysozyme-mediated lysis and digestion with Proteinase K was followed. The RNase-Free DNase Set (Qiagen, Cat. No. 79254) was used for an on-column DNase digestion for 30 min prior to RNA elution.

The quality of the isolated RNA was determined with a Qubit® (1.0) Fluorometer (Life Technologies) and by running a RNA-nanochip on a Bioanalyzer 2100 (Agilent). Only those samples with a 260/280 nm ratio between 1.8 and 2.1 and a 28s/18s ratio within 1.5 – 2.0 were further processed. The TruSeq RNA Sample Prep kit v2 (Illumina, Cat. No. RS-122-2001) was used in the subsequent steps. Briefly, total RNA samples (1000 ng) were ribosomal-RNA-depleted using the Ribo-Zero™ Magnetic kit for bacteria (Epicentre, Cat. No. MRZMB126) and then reverse-transcribed into double-stranded cDNA. The cDNA samples were fragmented, end-repaired and polyadenylated before ligation of TruSeq adapters containing the barcode index for multiplexing. Fragments containing TruSeq adapters on both ends were selectively enriched by PCR. The quality and quantity of the enriched libraries were validated using the 2200 TapeStation system (Agilent) and quantitative PCR. The products were a smear with an average fragment size of approximately 260 bp. The libraries were normalized to 10 nM in 10 mM Tris-Cl, pH 8.5 at 25°C supplemented with 0.1% (v/v) Tween-20.

Samples were pooled to equimolar amounts and sequenced in a single lane. The TruSeq SR Cluster Kit v4-cBot-HS (Illumina, Cat. No. GD-401-3001) was used for cluster generation using 8 pM of pooled normalized libraries on the cBOT. Sequencing was performed on the Illumina HiSeq 2500 single end 126 bp using the TruSeq SBS Kit v4-HS (Illumina, Cat. No. FC-401-3001).

Bioinformatics

After sequencing, reads were analyzed using SUSHI [4], an NGS data analysis workflow management system developed at the Functional Genomics Center Zurich. First, reads were quality-checked with FastQC (Babraham Bioinformatics) and low-quality ends were clipped (5 bases from the start, 10 bases from the end). Trimmed reads were aligned and mapped to the reference genome and transcriptome of *E. coli* K-12 DH10 (FASTA and GTF files, respectively, downloaded from Ensembl) with Bowtie version 2.1 [5].

For the whole genome sequencing experiments, polymorphisms were detected using GATK version 2.2.0, following the recommended DNA-seq best practices [6], and introduced in the NCBI reference *E. coli* K12 MG1655 using the GATK tool *FastaAlternateReferenceMaker*. This new FASTA file was then used as the background to identify the variants between the individuals in the sample groups. In every case, polymorphisms were considered to pass the filter, if they showed at least 15-fold coverage and a minimum quality score of 50.

The Unified Genotyper was used with the following options: baq Gap open penalty (whole-genome analysis) set to 30; minimum consensus coverage to genotype indels set to 8 (default: 5); minimum depth set to 19; minimum base quality score and minimum variants phred score set to 15; minimum variant quality score set to 50.

Variants were annotated using snpEFF version 3.4 [7], and distribution of the reads across genomic isoform expression was quantified using the R package GenomicRanges [8] from Bioconductor Version 3.0.

For the transcriptome analysis, mapped reads for each annotated gene were counted using CountOverlaps in the Bioconductor package GenomicRanges [8]. The differentially expressed genes were identified with the Bioconductor package edgeR [9] where the raw counts were normalized using the TMM (trimmed mean of M values) method [10]. The sequencing reads and raw counts have been deposited in Gene Expression Omnibus of NCBI under accession number GSE109819.

Enrichment analyses of the gene-expression data were made using the web tools at BioCyc.org, in particular the EcoCyc Database [11] . SmartTables and Omics Dashboard [12] enrichment parameters were set to include results whose p-value were less than 0.05 applying a Fisher exact statistics algorithm. In addition, statistics analyses specially targeted for sigma factor enrichment were done with the free statistical computing environment R v. 3.4.3. [13] using the fisher.test command and the experimental sigma factor–gene interaction dataset from RegulonDB v. 9.0 [14]. In all cases, p-values were first false discovery rate (FDR)-adjusted, using the procedure of multiple hypothesis testing correction of Benjamini and Hochberg [15].

Quantitative real time PCR

The whole experiment was performed following the MIQE guidelines (minimum information for publication of quantitative real-time PCR experiments) [16]. Total RNA was extracted as described for RNA sequencing analysis. Isolated RNA was further treated with the TURBO DNA-free kit (Ambion, Cat. No. AM1907), to remove residual genomic DNA. The purity and integrity of RNA was evaluated by electrophoresis in an agarose gel and measuring the ratio of the absorbance at 260/280 nm on a Nanodrop spectrophotometer. The RNA concentration was estimated by using Quant-iT™ RiboGreen RNA Assay Kit (Invitrogen, Cat. No. R11490). Total RNA (1 µg) was reverse-transcribed to obtain cDNA with a SuperScript First-Strand Synthesis kit using random

hexamers (Invitrogen, Cat. No. 11904018). Primers were designed with Primer3 software [17] or obtained from PrimerBank [18].

The quantitative PCR was performed in a Mx3005P qPCR System (Agilent) using 5 µl of 20-fold diluted cDNA product, the reagent SYBR Select Master Mix (Applied Biosystems, Cat. No. 4472908) and 10 pmol of specific primers for each gene in a 20 µl reaction volume. The temperature profile was 95°C for 2 min, followed by 40 cycles of 95°C for 15 s and 60°C for 1 min. A post-amplification melting-curve analysis was done to discard primer-dimer artifacts and to ensure reaction specificity by heating the products to 95°C for 5 s, followed by cooling to 60°C and heating to 95°C while monitoring fluorescence. PCR products of the correct lengths were verified by agarose gel electrophoresis. Samples without reverse transcriptase treatment were measured in parallel to determine the concentration of any contaminating DNA.

For each strain, three biological replicates were analyzed and three technical replicates were carried out for each qPCR measurement. The cycle threshold (CT) and efficiency values obtained were used for further analysis and calculation of relative expression levels using the 2ΔΔCt method [19]. Each sample was normalized using TATAA Universal RNA Spike II (TATAA Biocenter AB) as a spike–in internal control, and then the results from samples X and Y were compared to those in Z, as a calibrator sample. Tests for enzymatic inhibition and RNA extraction yield were performed as suggested for the TATAA Universal RNA Spike II (TATAA Biocenter AB).

Supplementary Figures and Tables

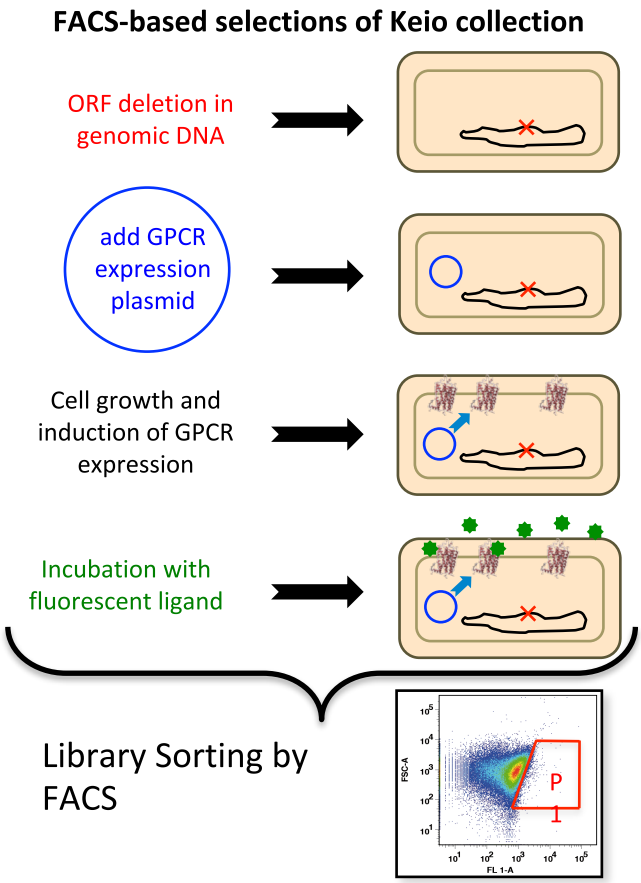

Figure S1: Scheme of selection and sorting process of the Keio mutants according to their GPCR expression.

The Keio clones were transformed with a GPCR-encoding plasmid (NTR1), the mutant strains were grown and GPCR expression was induced. The outer cell membrane was then permeabilized and functional receptors become labeled when the fluorescent ligands binds. *E. coli* cells were sorted by FACS to enrich for highly expressing mutants.

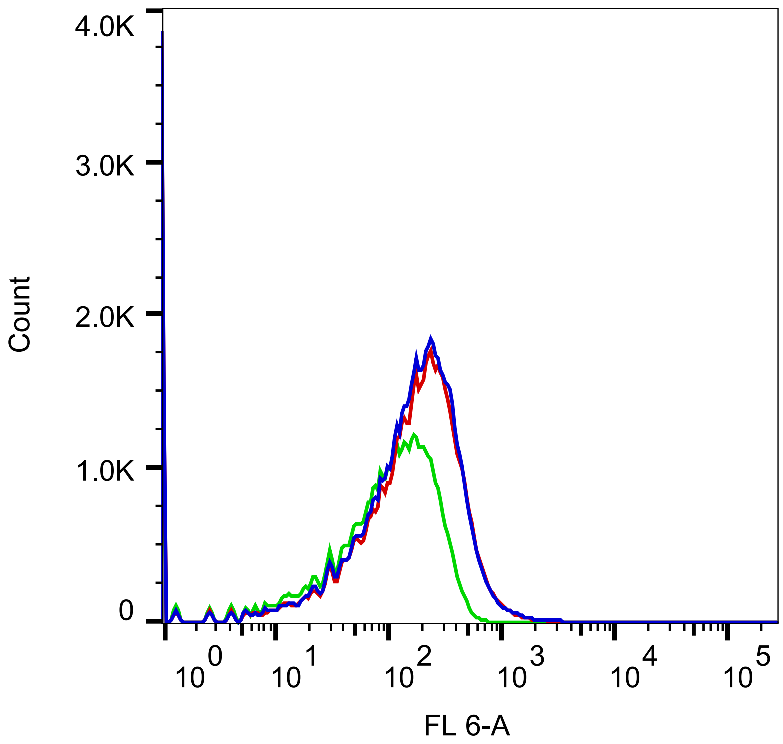

Figure S2: Gaussian distribution of the fluorescence signal of the mixed population as detected during the FACS selection.

Fluorescence signal after six iterative rounds of FACS selection. In green is shown the background, in red the *E. coli* BW25113 reference strain, in blue the library of the Keio collection with the NTR1 receptor expressed at 20°C.

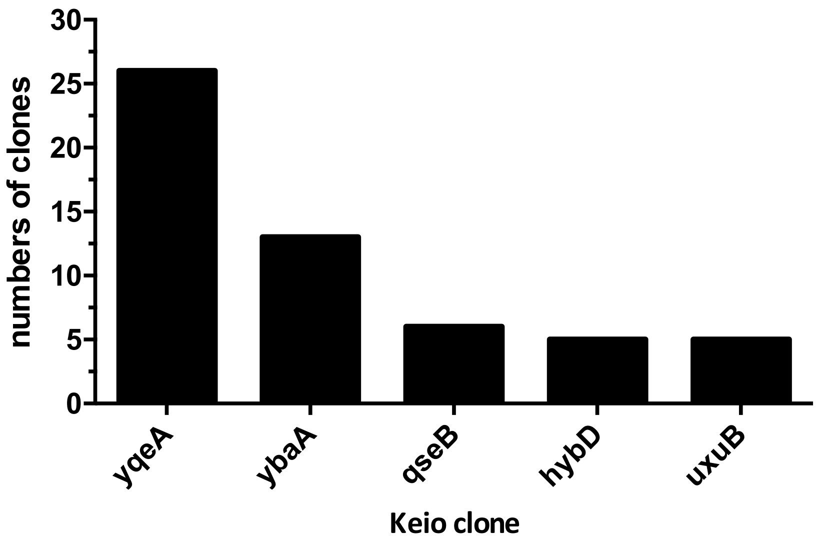

Figure S3: Keio clones enriched and identified by inverse PCR analysis.

100 clones were randomly picked from the pool of clones after six iterative rounds of FACS selection. Gene deletions were identified by iPCR. Only Keio clones detected more than once were taked in account and included in the figure. All genes named are gene deletions as in the Keio collection. Gene functions: *yqeA*, carbonate kinase homolog (function unknown); *ybaA*, function unknown; *qseB*, quorum sensing; *hybD*, maturation endoprotease for Ni-containing hydrogenase 2; and *uxuB*, D-mannonate oxidoreductase.

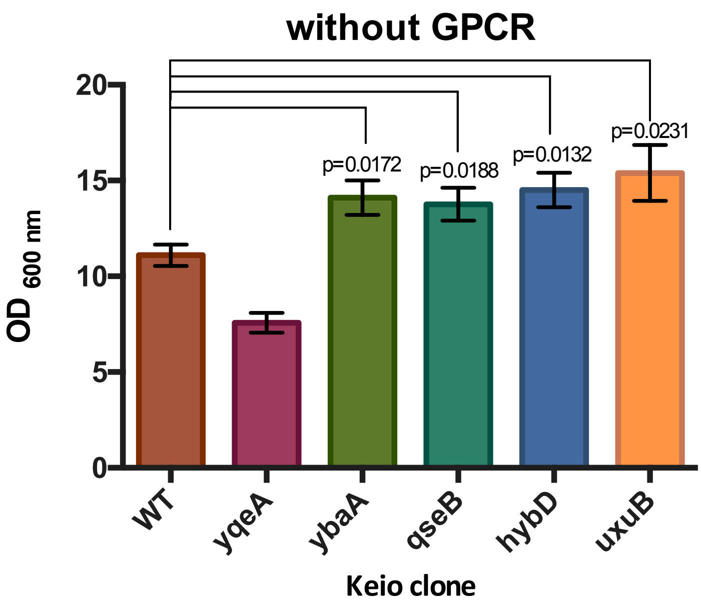

Figure S4: Selected Keio clones grow better at 20°

Characterization of the growth behavior of *E. coli* strain BW25113 (WT) and the most abundant clones of the selected Keio clones. Growth in rich medium (2xYT medium) was estimated with OD_{600nm} measurement after 20 hours of GPCR expression at 20ºC.

The x-axis label indicates the gene that is deleted on the respective Keio clone. Means and standard deviations from three independent experiments are shown.

p values are indicated for strains with statistically significant increases in growth versus wild-type *E. coli* BW25113 as calculated by two-tailed paired *t* test.

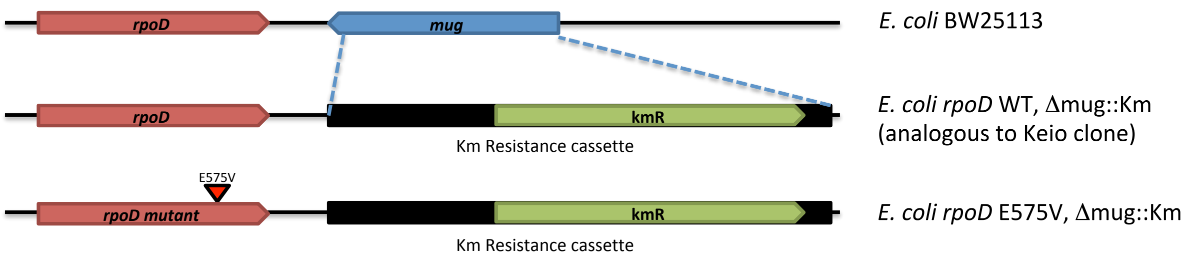

Figure S5: Genetic organization of the surrounding of the *rpoD* gene in *E. coli* BW25113.

The *rpoD* gene is shown in red. The kanamycin resistance cassette (black) with the KmR ORF (green) replaces the *mug* gene (blue) in new constructs. Details of the construction are summarized in the Supplementary Methods, section Site-directed mutagenesis in the *E. coli* genome**.**

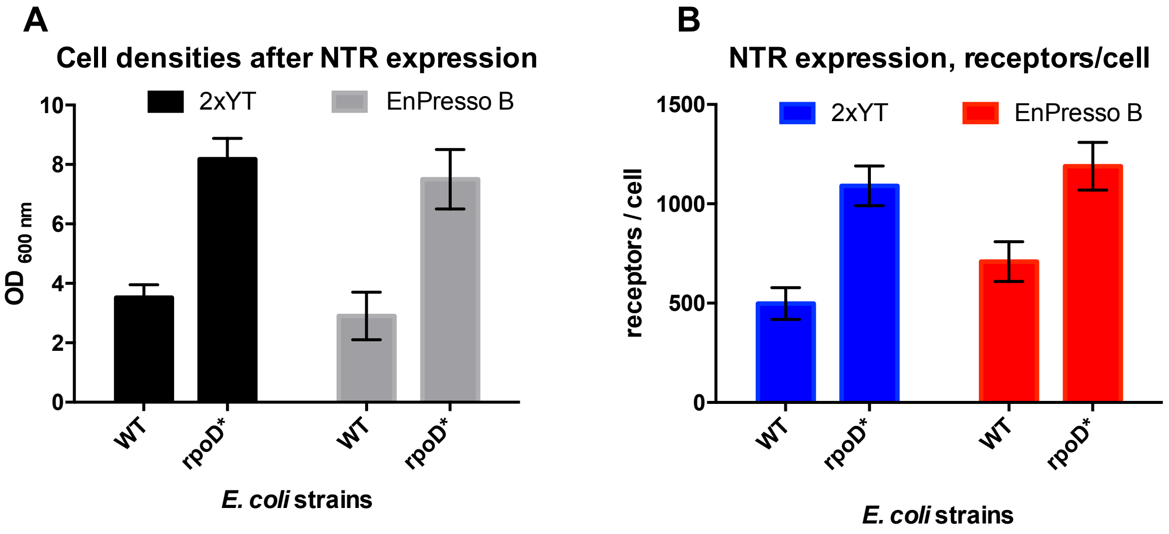

Figure S6: NTR1 expression in EnPresso medium.

E. coli wt BW25113 and the *rpoD* mutant strains were transformed with the plasmid pRG-NTR. Characterization was done in 2xYT rich medium and EnPressoB optimized medium for slow glucose feeding. Means and standard deviations from three independent experiments are shown. (A) Growth was estimated with OD_{600nm} measurements after 20 hours of NTR1 expression at 20°C and (B) the receptor expression levels were assessed by radioligand binding assays.

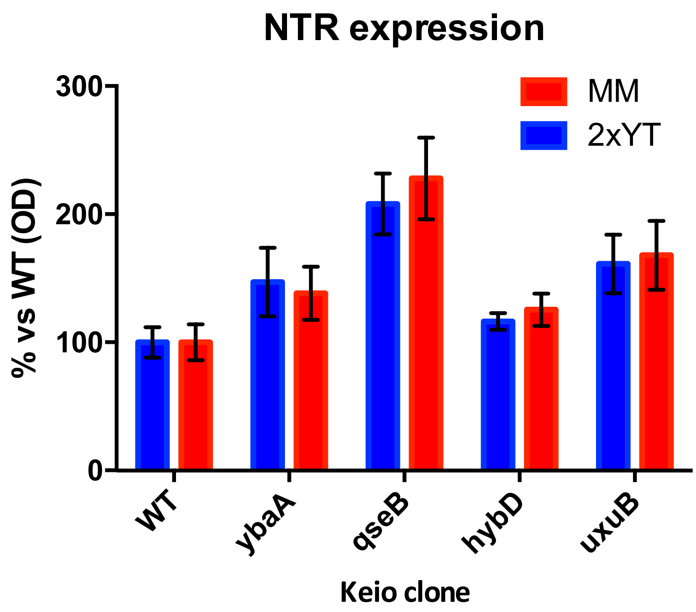

Figure S7: Growth at 20°C during NTR1 expression in M9 minimal medium (MM) compared with rich medium (2xYT).

E. coli strain BW25113 (wt) and 4 clones of the selected Keio clones harboring the plasmid pRG-NTR were grown in M9 minimal medium (MM). Growth was estimated with OD_{600nm} measurements after 20 hours of GPCR expression at 20°C. Results are normalized to values for the *E. coli* wt strain. Results of growth in rich medium 2xYT as in Figure S3 were included for comparison. The x-axis label indicates the gene that is deleted on the respective Keio clone. Means and standard deviations from three independent experiments are shown.

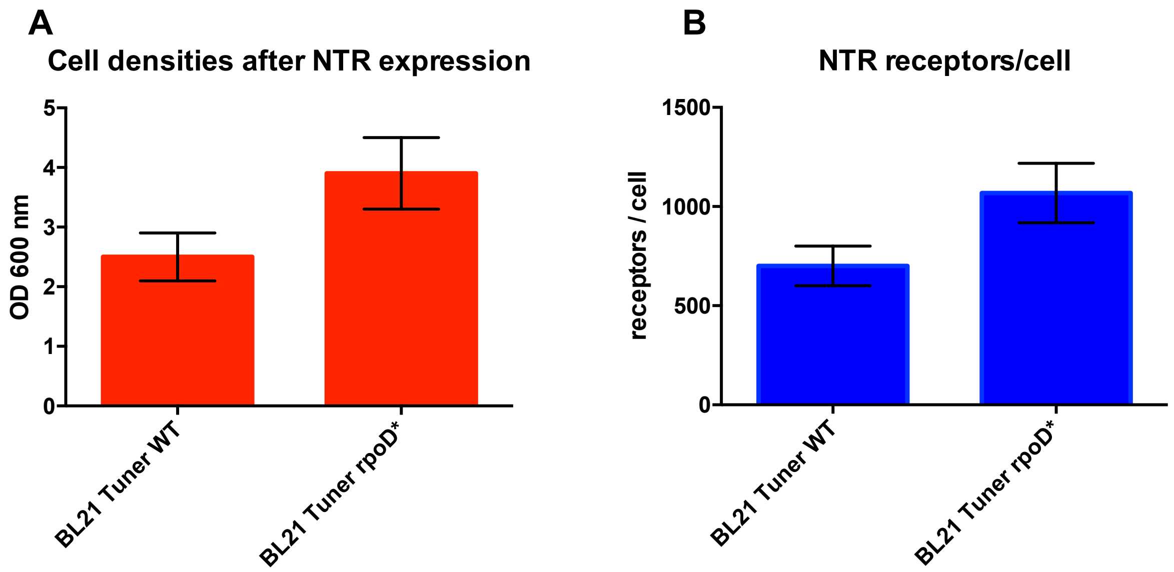

Figure S8: Expression of NTR1 in *E. coli* BL21.

E. coli BL21 Tuner and the *rpoD* mutant BL21 Tuner strains were transformed with the pRG-NTR plasmid. (A) Growth in 2xYT rich medium was estimated with OD_{600nm} measurement after 20 hours of NTR1 expression at 20°C and (B) the receptor expression levels were assessed by radioligand binding assays. Means and standard deviations from three independent experiments are shown.

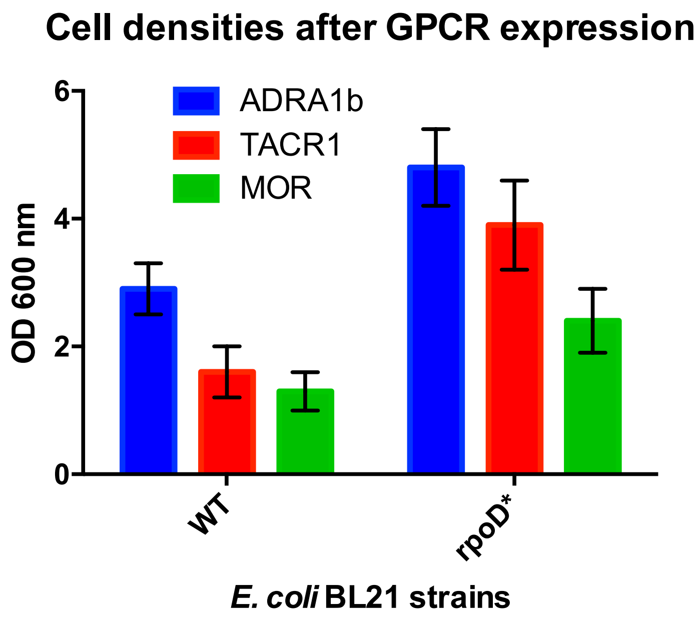

Figure S9: Growth of *E. coli* BL21 strains expressing ACRA, TACR, MOR receptors.

E. coli BL21 Tuner and the *rpoD* mutant BL21 Tuner strains were transformed with pRG plasmid derivatives encoding the wild-type version of ADRA1b, TACR1 and MOR GPCRs. Growth was estimated with OD_{600nm} measurements after 20 hours of GPCR expression at 20°C. Means and standard deviations from three independent experiments are shown.

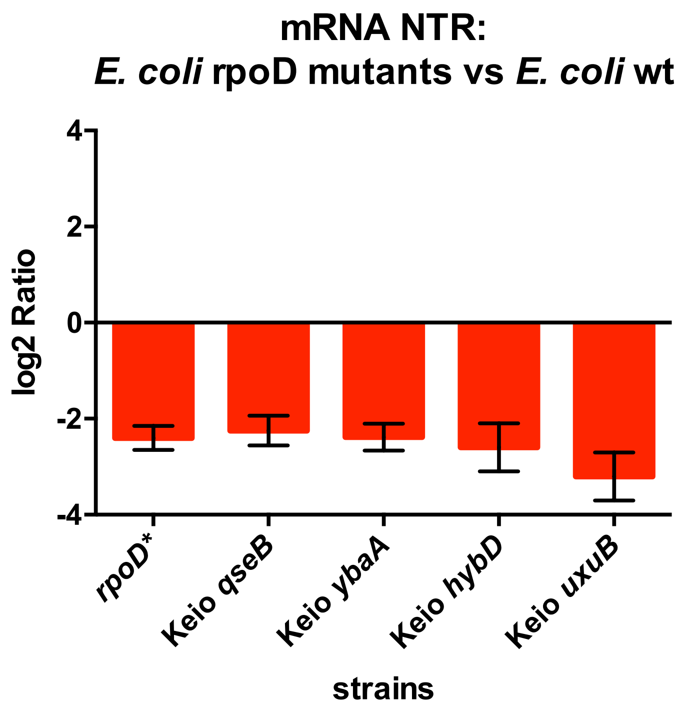

Figure S10: Expression of the NTR1 gene in strains with mutations in the *rpoD* gene.

The newly constructed *E. coli rpoD* mutant and the 4 selected Keio clones with *rpoD* mutations were transformed with the pRG-NTR plasmid. Expression of the NTR1 gene was tested by using quantitative real-time PCR and normalized with TATAA Universal RNA Spike II. Results are shown as log_2 ratio versus value of NTR1 expression in *E. coli* BW25113 wt. Means and standard deviations from three independent experiments are shown.

		E. coli rpoD vs *E. coli* WT		*E. coli* WT (NTR) vs *E. coli* WT		*E. coli* rpoD (NTR) vs *E. coli WT (NTR)*	
**Panels**	**Subsystems**	Up	Down	Up	Down	Up	Down
**Biosynthesis**	Amino Acid Biosynthesis					0.678	
	Nucleosides and Nucleotides Biosynthesis				0.356	1.14	
	Fatty Acid and Lipid Biosynthesis						
	Amines and Polyamines Biosynthesis						
	Carbohydrates Biosynthesis		0.732				
	Secondary Metabolites Biosynthesis						
	Cofactors, Prosthetic Groups, Electron Carriers Biosynthesis						
	Cell Structures Biosynthesis						
	Metabolic Regulators Biosynthesis						
	Other Biosynthesis						
**Degradation**	Amino Acid Degradation		1.13		0.36		
	Nucleosides and Nucleotides Degradation						
	Fatty Acid and Lipids Degradation	0.405	1.13			0.316	
	Amines and Polyamines Degradation		1.5				
	Carbohydrates and Carboxylates Degradation		0.66				
	Secondary Metabolites Degradation						
	Alcohols Degradation	2.01		0.082			
	Aromatic Compounds Degradation						
	Polymeric Compounds Degradation						
	Other Degradation				0.323		
**Energy**	Glycolysis			1.15			
	TCA cycle						
	Pentose Phosphate Pathway						
	Fermentation			1.14			
	Aerobic Respiration						
	Anaerobic Respiration			4.25			0.91
	Other Energy						
**Central Dogma**	Transcription Proteins			0.584			
	Translation Proteins						
	DNA Metabolism						
	RNA Metabolism						
	Protein Metabolism						
	Protein Folding and Secretion						
**Regulation**	Signal transduction pathways						
	Sigma Factors						
	Sigma Factor Regulons		1.95				
	Transcription Factors						
	Transcription Factor Regulons		5.85	2.89		0.456	1.32
**Cell Exterior**	Transport Proteins						
	Cell Wall Biogenesis/Organization Proteins						
	Lipopolysaccharide Metabolism Proteins				0.924		
	Pilus Proteins						
	Flagellar Proteins				10.5	5.3	
	Outer Membrane Proteins						
	Plasma Membrane Proteins	1.55			2.39		
	Periplasmic Proteins						
	Cell Wall Component Proteins						
**Response to Stimulus**	Starvation		0.705				
	Heat						
	Cold				0.975		
	DNA Damage						
	Osmotic Stress		0.93				
	pH						
	Detoxification						
	Oxidant Detoxification			0.123			
	Other Proteins involved in Stimulus Response						

Figure S11: Summary of gene enrichment analysis using Pathway Tools Omics Dashboard [12] with the RNA-seq data. Numbers are an enrichment score: -log10(p-value), where p-values were computed using Grossmann's parent-child-union variation of the Fisher-exact test, and applying the specified multiple hypothesis correction. Analyses were done using subsets of up- or down-regulated genes in each comparison.

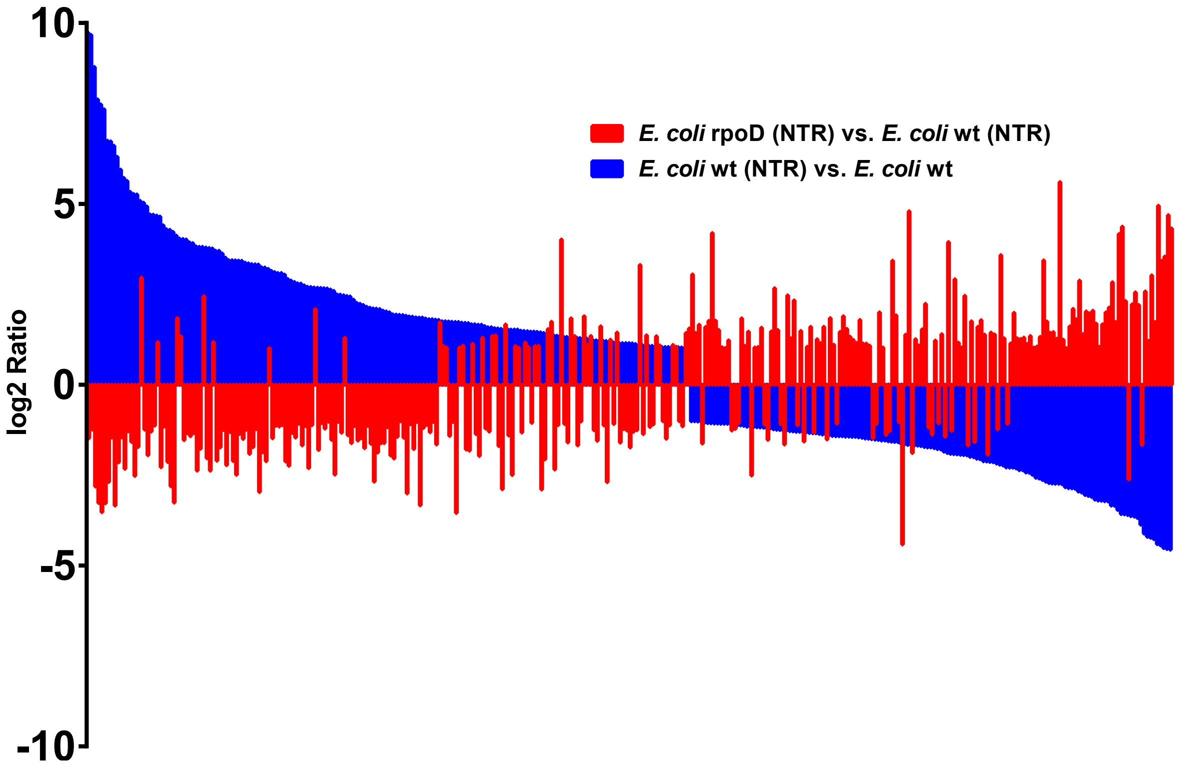

Figure S12: Comparison of differentially expressed genes.

RNA-seq data (see Table S1 for the full set of data) were used to analyze the pattern of global gene expression in the different *E. coli* strains. In comparing *E. coli* BW25113 harboring pRG-NTR versus *E. coli* BW25113 (without NTR), $\log_2$ ratios of gene expression are shown in blue in a descending order from left to right. Only those genes with log2 ratio bigger than 1 or smaller than -1 are plotted. In the same gene order, $\log_2$ ratios of gene expression are shown in red when comparing *E. coli rpoD* mutant harboring pRG-NTR versus *E. coli* BW25113 harboring pRG-NTR.

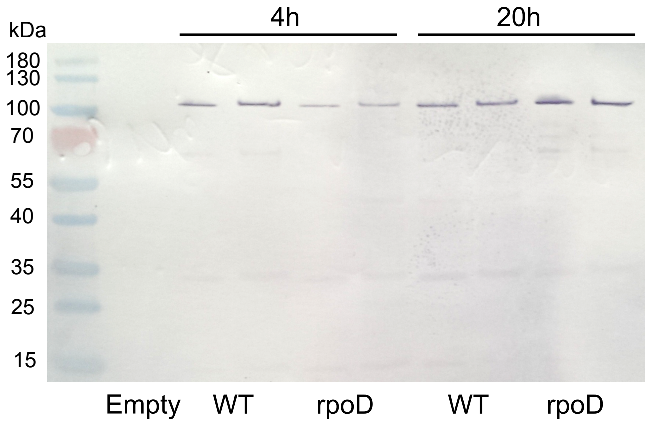

Figure S13: Full-size blot image of Western blot presented in Figure 5.

NTR1 protein levels were monitored with western blots using an anti-MBP antibody (in duplicates).

Table S1: Spreadsheet with RNA Seq data (separate file)

Table S2: Summary of differentially expressed genes:

Strains compared	p-value<0.01	Up-regulated	Down-regulated
rpoD vs. wt*	1534	164	353
wt NTR vs. wt	1654	665	548
rpoD NTR * vs. rpoD**	2429	1020	416
rpoD NTR vs. wt NTR*	2197	334	294

Differentially expressed genes are counted with p-value <0.01, log_2ratio >1 (up-regulated) or <-1 (down-regulated) and at least 10 counts for each *E. coli* gene.

Table S3: Statistical analysis of sigma factor regulons of up- and down-regulated genes

	subtotal	s70		s24		s32		s54		s28		s38		s19		total known
**Total, normal distribution**		1444		459		262		134		153		257		5		2597
			p Value		*p Value*		*p Value*		*p Value*		*p Value*		*p Value*		*p Value*	
**rpoD*_20 vs WT_20 total**		584		125		89		36		40		135		5		1014
**rpoD*_20 vs WT_20 UP**	164	71	*2.46E-02*	12	*0.5198*	11	*0.2742*	1	*0.9875*	2	*0.9763*	12	*0.5696*	2	*3.70E-02*	
**rpoD*_20 vs WT_20 DOWN**	353	138	*0.2171*	34	*0.5332*	17	*0.8002*	9	*0.806*	4	*0.9868*	70	***2.20E-16***	0	*1*	
**WT_NTR_20 vs WT_20 total**		578		170		107		54		62		129		5		1105
**WT_NTR_20 vs WT_20 UP**	665	233	*0.6641*	61	*0.7378*	50	***3.63E-02***	35	***7.39E-05***	11	*0.9998*	58	***3.51E-02***	0	*1*	
**WT_NTR_20 vs WT_20 DOWN**	548	199	*0.1885*	56	*0.4272*	23	*0.9935*	11	*0.9878*	35	***1.88E-03***	43	*0.2719*	4	***2.60E-02***	
**rpoD*_NTR_20 vs WT_NTR_20 total**		797		212		141		55		66		131		5		1407
**rpoD*_NTR_20 vs WT_NTR_20 UP**	334	156	***2.95E-07***	24	*0.9873*	18	*7.45E-01*	6	*0.9481*	19	***4.21E-02***	12	*0.98*	5	***3.65E-04***	
**rpoD*_NTR_20 vs WT_NTR_20 DOWN**	294	80	*0.9916*	37	*0.1013*	19	*0.3093*	14	*0.105*	1	*1*	34	***5.78E-04***	0	*1*	
**rpoD*_NTR_20 vs rpoD*_20 Total**		883		218		143		74		72		149		3		1542
**rpoD*_NTR_20 vs rpoD*_20 UP**	1001	364	*0.1566*	92	*0.6221*	59	*0.5098*	54	***6.39E-05***	24	*0.9852*	81	***1.72E-02***	0	*1*	
**rpoD*_NTR_20 vs rpoD*_20 DOWN**	416	145	*0.4173*	41	*0.4671*	20	*0.8716*	1	*1*	16	*0.1858*	10	*0.9999*	1	*0.4623*	

Differentially expressed genes: p-value<0.01, log_2ratio >1 (up-regulated) or <-1 (down-regulated) and at least 10 counts for each *E. coli* gene.

REFERENCES

1. Horton, R. M. *et al.* Gene splicing by overlap extension. *Meth. Enzymol.* **217,** 270–279 (1993).
2. Datsenko, K. A. & Wanner, B. L. One-step inactivation of chromosomal genes in Escherichia coli K-12 using PCR products. *Proc Natl Acad Sci U S A* **97,** 6640–6645 (2000).
3. Heermann, R., Zeppenfeld, T. & Jung, K. Simple generation of site-directed point mutations in the Escherichia coli chromosome using Red(R)/ET(R) Recombination. *Microb Cell Fact* **7,** 14 (2008).
4. Hatakeyama, M. *et al.* SUSHI: an exquisite recipe for fully documented, reproducible and reusable NGS data analysis. *BMC Bioinformatics* **17,** 228 (2016).
5. Langmead, B. & Salzberg, S. L. Fast gapped-read alignment with Bowtie 2. *Nat Meth* **9,** 357–359 (2012).
6. DePristo, M. A. *et al.* A framework for variation discovery and genotyping using next-generation DNA sequencing data. *Nat. Genet.* **43,** 491–498 (2011).
7. Cingolani, P. *et al.* A program for annotating and predicting the effects of single nucleotide polymorphisms, SnpEff: SNPs in the genome of Drosophila melanogaster strain w1118; iso-2; iso-3. *Fly (Austin)* **6,** 80–92 (2012).
8. Lawrence, M. *et al.* Software for computing and annotating genomic ranges. *PLoS Comput Biol* **9,** e1003118 (2013).
9. Robinson, M. D., McCarthy, D. J. & Smyth, G. K. edgeR: a Bioconductor package for differential expression analysis of digital gene expression data. *Bioinformatics* **26,** 139–140 (2010).
10. Robinson, M. D. & Oshlack, A. A scaling normalization method for differential expression analysis of RNA-seq data. *Genome Biol.* **11,** R25 (2010).
11. Keseler, I. M. *et al.* The EcoCyc database: reflecting new knowledge about Escherichia coli K-12. *Nucleic Acids Res* **45,** D543–D550 (2017).
12. Paley, S. *et al.* The Omics Dashboard for interactive exploration of gene-expression data. *Nucleic Acids Res* **45,** 12113–12124 (2017).
13. Team, R. C. R: A Language and Environment for Statistical Computing. (2017). Available at: https://www.R-project.org/. (Accessed: 21st March 2018)
14. Gama-Castro, S. *et al.* RegulonDB version 9.0: high-level integration of gene regulation, coexpression, motif clustering and beyond. *Nucleic Acids Res* **44,** D133–43 (2016).
15. Benjamini, Y. & Hochberg, J. Controlling the false discovery rate: a practical and powerful approach to multiple testing. *JSTOR* **57,** 289–300 (1995).
16. Bustin, S. A. *et al.* The MIQE Guidelines: Minimum Information for Publication of Quantitative Real-Time PCR Experiments. *Clin Chem* **55,** 611–622 (2009).
17. Untergasser, A. *et al.* Primer3--new capabilities and interfaces. *Nucleic Acids Res* **40,** e115–e115 (2012).
18. Wang, X., Spandidos, A., Wang, H. & Seed, B. PrimerBank: a PCR primer database for quantitative gene expression analysis, 2012 update. *Nucleic Acids Res* **40,** D1144–9 (2012).
19. Livak, K. J. & Schmittgen, T. D. Analysis of Relative Gene Expression Data Using Real-Time Quantitative PCR and the 2−ΔΔCT Method. *Methods* **25,** 402–408 (2001).
